# Regulation of reticular adhesions by KANK2 and talin2 in two melanoma cell lines

**DOI:** 10.1186/s12964-026-02904-1

**Published:** 2026-04-24

**Authors:** Anja Rac, Marija Lončarić, Nikolina Stojanović, Mahak Fatima, Mirna Rešetar, Dalibor Hršak, Jonathan D. Humphries, Martin J. Humphries, Andreja Ambriović-Ristov

**Affiliations:** 1 Laboratory for Cell Biology and Signalling, Division of Molecular Biology, Ruđer Bošković Institute, Zagreb, Croatia; 2 Manchester Cell-Matrix Centre, Faculty of Biology, Medicine & Health, University of Manchester, Manchester, UK; 3 Laboratory for Computational Biology and Translational Medicine, Division of Electronics, Ruđer Bošković Institute, Zagreb, Croatia; 4 Department of Life Science, Manchester Metropolitan University, Manchester, UK

**Keywords:** Melanoma cells, Reticular adhesions, Talin2, KANK2, Integrin αVβ5, Adhesome

## Abstract

**Background:**

Integrins bind extracellular matrix proteins and, upon clustering, form multimolecular integrin adhesion complexes (IACs) that connect to and regulate the cell cytoskeleton, influencing various aspects of cell behaviour. Alongside well-characterized focal adhesions (FAs) and fibrillar adhesions (FBs), a new class of IACs, reticular adhesions (RAs), have been identified. RAs, formed by integrin αVβ5, lack actin association and classical FAs markers and their physiological role in cells remains poorly understood. Previously, we showed that two melanoma cell lines, MDA-MB-435S and RPMI-7951, preferentially use integrin αVβ5 (which can form FAs or RAs) for adhesion under long-term culture conditions. Here we investigate the composition of RAs in these two melanoma cell lines.

**Methods:**

Cells were treated with the actin polymerisation inhibitor cytochalasin D to disrupt FAs and enable isolation and mass spectrometry–based analysis of RAs components. Western blotting, immunofluorescence microscopy and proximity ligation assays (PLA) were performed to assess protein expression and localization within RAs. The effects of talin2 and KN motif and ankyrin repeat domains protein 2 (KANK2) knockdown on RA composition were examined in both melanoma cell lines.

**Results:**

Known RA-associated proteins, including the Adaptor protein 2 (AP2) complex, disabled homolog 2 (DAB2), Numb and talin2 were identified in both lines, along with a new protein, KANK2. PLA following actin disruption confirmed the proximity of KANK2 and talin2 in RAs. Knockdown experiments demonstrated that although both talin2 and KANK2 are located in RAs, neither is essential for RA formation. Talin2 knockdown resulted in reduced abundance of RA components in both cell lines. In MDA-MB-435S cells, KANK2 knockdown produced a similar effect. However, in RPMI-7951 cells, KANK2 knockdown had no significant effect on RA components abundance, reflecting the differential localization and role of KANK2 in this cell line.

**Conclusion:**

We provide a comprehensive analysis of RAs in two melanoma cell lines and identify KANK2 as a novel RA-associated protein. The differential effects of KANK2 knockdown on the abundance of RAs underscores its distinct role in αVβ5-mediated adhesions, FAs and RAs, across the two cell lines. These findings emphasize the importance of adhesion crosstalk in regulating integrin αVβ5–mediated cell adhesions.

**Supplementary Information:**

The online version contains supplementary material available at 10.1186/s12964-026-02904-1.

## Abstract (plain language summary)

Cells attach to their surroundings through structures called adhesions. Integrin receptor activation initiates the formation of integrin adhesion complexes at the cell membrane regulating various aspects of normal and tumour cell behaviour. In addition to well-known adhesion types, a new kind, reticular adhesions, has recently been discovered. These adhesions are formed by the integrin αVβ5, do not connect to the actin cytoskeleton, and differ in composition from focal adhesions. In this study, we examined reticular adhesions in two melanoma cell lines, MDA-MB-435S and RPMI-7951, which both preferentially use integrin αVβ5 for adhesion, forming focal and reticular adhesions, but differ in their ability to form fibrillar adhesions. To analyse their composition, we disrupted the actin cytoskeleton using cytochalasin D, isolated reticular adhesions and analysed them using mass spectrometry-based proteomics. Along with known reticular adhesion proteins, we identified a new protein, KN motif and ankyrin repeat domains protein 2 (KANK2), within these structures, localised in close proximity to talin2. While neither protein is essential for reticular adhesion formation, reducing talin2 levels decreased the abundance of reticular adhesion components in both cell lines. Reducing KANK2 had distinct effects: it reduced the abundance of reticular adhesions components in MDA-MB-435S cells but had little impact in RPMI-7951 cells. The differential effects of KANK2 knockdown underscores its differing roles in αVβ5-mediated focal adhesions across the two cell lines. These findings underscore the importance of adhesion crosstalk in regulating integrin αVβ5–mediated cell adhesions.

**Supplementary Information:**

The online version contains supplementary material available at 10.1186/s12964-026-02904-1.

## Introduction

Most cells in multicellular organisms require attachment to their surroundings through cell-cell or cell-extracellular matrix (ECM) adhesions. In cell-matrix adhesions, integrins, a family of transmembrane receptors, have long been recognized as the central scaffolds around which these complexes are built. Through binding to the ECM and clustering, integrins form multimolecular adhesion complexes [1]. The composition of integrin adhesion complexes (IACs) varies depending on the type of adhesions they form. Focal adhesions (FAs) mature from nascent adhesions and facilitate force transduction through tight links to contractile actomyosin fibers [2, 3]. Formed by many integrin heterodimers, FAs are located primarily at the cell edge. Fibrillar adhesions (FBs), formed by integrin α5β1 [4], are located in the cell centre of the migrating cell [5, 6]. FBs arise through translocation of fibronectin-bound α5β1 integrins from the periphery to the centre [6, 7] in a tension-dependent manner and have a role in fibronectin fibrillogenesis [8, 9]. Integrins are also components of adhesion structures that lack strong actomyosin connections, are enriched in endocytic proteins [10, 11], yet still function as signalling platforms [12, 13]. These structures, formed as a result of frustrated endocytosis [14–16], and are referred to as reticular adhesions (RAs) [10], flat clathrin lattices [11, 12, 14, 17], or clathrin plaques [18–21]. RAs are mediated exclusively by integrin αVβ5 but lack most IAC components, except talin2 and tensin3 [10]. Recent studies have shown that RAs are larger than flat clathrin lattices and contain them in an interspersed manner [22]. Unlike FAs, RAs persist throughout cell division to enable effective mitosis and also transmit spatial memory from pre-mitotic to post-mitotic daughter cells [10, 23]. Since RAs form through arrested endocytosis, their core components include proteins involved in clathrin-mediated endocytosis, such as adaptor protein 2 (AP2), disabled protein 2 (Dab2), NUMB Endocytic Adaptor Protein (Numb), Epidermal Growth Factor Receptor Pathway Substrate 15 (EPS15), Epidermal Growth Factor Receptor Pathway Substrate 15 Like 1 (EPS15L1), intersectin (ITSN), and Low Density Lipoprotein Receptor Adaptor Protein 1 (LDLRAP1 or ARH) [10, 11, 14, 24]. None of these proteins are present exclusively in RAs, and therefore cannot be used as markers. Lukas et al. [25] have recently identified stonin1 as a protein exclusively present at RAs. However, they emphasized that stonin1 is not ubiquitously expressed.

Talins are key cytoplasmic mechanosensitive proteins that mediate binding of integrins to the cytoskeleton [26]. There are two different isoforms of talin: talin1 and talin2. Talin1 forms the core of FAs, increasing the affinity of integrin for ligands (integrin activation) and recruiting a range of proteins [27, 28]. Abrogation of talin1 expression leads to FA disruption, while talin2 has been shown to influence FA dynamics [29, 30]. Talin2 is mainly found at large FAs and FBs [31, 32] but also in RAs [10]. Talins directly bind to actin and coordinate microtubule (MT) recruitment to adhesion sites via interaction with KANK proteins [26, 29, 33–35].

KANK2, a member of the KANK (kidney ankyrin repeat-containing) family proteins that bind the talin rod through a KN motif, is mainly found in mesenchymal cells [36]. Along with KANK1, KANK2 localizes to the rims of mature integrin-containing FAs and adjacent regions called cortical microtubule stabilizing complexes (CMSCs). The CMSC, recruited via KANK2-talin1 interaction [34] or talin2-KANK2 interaction [29] stabilizes MTs near mature FAs and regulates actin-microtubule crosstalk. In some cells, behind the lamella, KANK2 binding to talin promotes integrin activation, and at the same time diminishes F-actin binding to talin. Consequently, this leads to the translocation of β1 integrins into FBs [34]. FBs contain talin2 [31] and KANK2 [30, 32].

RAs have been observed in various cell lines [10, 11, 25, 37]; however, information on their molecular composition has mostly come from U2OS cells [10], keratinocytes [11], and C2C12 cells [25]. Here, a comprehensive analysis of RAs in two melanoma cell lines MDA-MB-435S and RPMI-7951, which differ in their ability to form FBs in long-term culture [30], is presented. In RAs of both cell lines, previously known RA proteins, including talin2 [10], were identified, as was KANK2. Talin2 and KANK2 were not essential for RA formation in any of the analysed cell lines. Talin2 knockdown reduced the abundance of RA components in both cell lines. Surprisingly, KANK2 knockdown produced different effects on RAs depending on its preferential localization and function in the cells. In the MDA-MB-435S cell line, where KANK2 functionally interacts with talin2 in FAs [29], its knockdown produced a similar effect on RAs as talin2 knockdown. However, in RPMI-7951 cells, where KANK2 is present in FAs but also localized in FBs where it has specific function in cell migration [30], KANK2 knockdown did not affect the abundance of RA components. Our results show that talin2 and KANK2 knockdown affects RA component abundance depending on the role of these molecules within αVβ5 FAs, indicating adhesion crosstalk.

## Materials and methods

### Cell cultures

The human melanoma cell lines MDA-MB-435S and RPMI-7951 were obtained from the American Type Culture Collection (ATCC). Cells were grown on uncoated surfaces in high-glucose DMEM containing sodium bicarbonate and L-glutamine without sodium pyruvate (Sigma-Aldrich) supplemented only with 10% (v/v) FBS (Invitrogen) (DMEM-FBS) with no added antibiotics, at 37 °C with 5% CO2 (v/v) in a humidified atmosphere.

### Transient siRNA transfection

For transient siRNA transfection experiments, cells (MDA-MB-435S, 4 × 10^4^, 4 × 10^5^ or 1.7 × 10^6^; RPMI-7951, 3.7 × 10^4^, 5 × 10^5^ or 1 × 10^6^) were plated in 24-well, 6-well cell culture plates or 10 cm diameter Petri dishes to achieve 60–80% confluence after 24 h. Cells were transfected 24 h later, using Lipofectamine RNAiMax (13778150, Thermo Fisher Scientific), with 25 nM of control (Silencer™ Select Negative Control No. 1 siRNA, Ambion), ITGA5 (s7547, Ambion), ITGB5 (s7591, Ambion) or gene-specific siRNA for KANK2 (target sequence: ATGTCAACGTGCAAGATGA), TLN2 (target sequence: TTTCGTTTTCATCTACTCCTT) [29], all purchased from Sigma. Knockdown was validated by SDS-PAGE and western blotting (WB) using specific antibodies and matched labelled secondary antibodies (See Supplementary Table S1, Supplementary Material 1).

### Cell treatment

To enrich samples with RA proteins, cells were treated with different concentrations (1, 4, 7, 20 nM in DMSO) of actin polymerization inhibitor cytochalasin D (CytoD) for 2 h at 37 °C. Control cells were treated with equivalent amount of DMSO. Cells were then further processed for Mass Spectrometry (MS), WB or immunofluorescence (IF) analysis.

### Isolation of IACs

IACs were isolated from cells cultivated in 10 cm diameter Petri dishes for 48 h post-transfection for MDA-MB-435S cells or 72 h post-transfection for RPMI-7951, with (to isolate RAs) or without CytoD treatment (to isolate all adhesions), as described previously [29, 37, 38]. Briefly, cells were washed with DMEM-HEPES and incubated with Wang and Richard’s reagent (DTBP, 6 mM, Thermo Fisher Scientific) for 10 min (MDA-MB-435S) or 15 min (RPMI-7951). DTBP is a membrane-permeable, thiol-cleavable crosslinker that covalently links proximal proteins within adhesion sites. This crosslinking step stabilizes protein–protein interactions within IACs, preserving labile or transient associations during subsequent cell lysis and wash procedures allowing efficient enrichment of IAC components while minimizing their loss or dissociation. The optimal duration of crosslinking was determined empirically based on protocol optimization by [29, 30, 37], ensuring maximal preservation of IACs without excessive crosslinking, which would introduce cytoplasmic or nuclear proteins into the samples. DTBP was quenched with 0.03 M Tris-HCl (pH 8) and cells were lysed using modified RIPA buffer (50 mM Tris-HCl, pH 7.6; 150 mM NaCl; 5 mM disodium EDTA, pH 8; 1% (w/v) Triton X-100, 0.5% (w/v) SDS, 1% (w/v) sodium deoxycholate). Cell bodies were removed by high-pressure washing with tap water for 10 s, and remaining IACs were collected by scraping into adhesion recovery solution (125 mM Tris-HCl, pH 6.8; 1% (w/v) SDS; 150 mM dithiothreitol). Samples containing isolated IACs were acetone-precipitated and further processed for MS or WB analysis [39].

### Sample preparation for MS, data analysis, protein-protein interaction network analysis and functional enrichment analysis

For MS analysis, isolated IAC samples were prepared using in-gel trypsin digestion [37, 38], and analysed using a modified version of the LC-MS/MS method, as previously described [40]. Samples were analysed by LC-MS/MS using an UltiMate 3000 Rapid Separation LC (RSLC, United States) coupled to Thermo QE HF with U3000 nanoUPLC mass detector (Thermo Fisher Scientific, United States) with electrospray ionization. Peptide mixtures were eluted for 60 min. To identify proteins after MS analysis, data were searched against the human Swissprot and Trembl database (July 2022, July 2023) using Mascot (Matrix science, version 2.5.1). Fragment ion tolerance was set to 0.02 Da, while parent ion tolerance was 5 PPM. We set the carbamidomethylation of cysteine as a fixed modification and oxidation of methionine as a variable modification. In further analysis, we considered only peptides with ions of charge precursors 2+, 3 + and 4+. Protein identifications were further refined using Scaffold (Proteome Software, version 5.1.0). Protein (99,9%) and peptide (90%) probabilities were assigned using the Protein Prophet algorithm [41] as incorporated by Scaffold including a minimum of four spectral counts per protein. Spectral counts were used as a measure of protein abundance. Data have been deposited in the ProteomeXchange Consortium [42] (dataset ID: PXD071922) via the PRIDE repository [43]).

Human protein–protein interactions (PPIs) were loaded from STRING database, using stringApp (confidence score cut-off = 0.40, maximum additional interactors = 0) [44] for Cytoscape software (version 3.7.1) [45]. QSpec statistical method [46] was used for MS data to measure the significance of differentially identified proteins in control cells and treated cells. For visualization of differentially expressed proteins, volcano plot (GraphPad Prism version 9.0.0 (GraphPad Software)) with the following setup was created: −Log (FDR) ≥ 0.05 corresponding to FDR ≤ 0.05; and fold change ≥ 1.5 or 2.

### Confocal microscopy

For IF analysis, cells were plated on coverslips in 24-well plates. After 48 h, cells were fixed using ice-cold methanol for 10 min or 2% (v/v) paraformaldehyde for 12 min followed by permeabilization with 0.1% (v/v) Triton X-100 for 2 min, and stained with protein-specific primary antibodies for 1 h, followed by incubation with conjugated secondary antibodies for 1 h. The primary and secondary antibodies are listed in Supplementary Table S1, Supplementary Material 1. Actin stress fibers were stained with rhodamine phalloidin (Cell Signaling Technology). Cells were mounted with DAPI Fluoromount-G (SouthernBiotech) and fluorescence and respective Interference reflection microscopy (IRM) images were acquired using an inverted confocal microscope (Leica TCS SP8 X, Leica Microsystems) with the HC PL APOCS2 63×/1.40 oil-immersion objective, zoom set at 2×. Images were analysed using LAS X software 3.1.1 (Leica Microsystems) and ImageJ. IRM images are included alongside each IF analysis to confirm that imaging was performed in the appropriate cellular layer containing the adhesion complexes, with the focus adjusted to the adhesion sites of cells on the upper surface of the glass coverslip.

### Proximity ligation assay

Proximity ligation assay (PLA), using Duolink^®^ PLA technology [47], was performed according to manufacturer’s instructions. Briefly, cells seeded on coverslips for 48 h were fixed, blocked and incubated with selected primary antibodies for 1 h at 37 °C in a preheated humidity chamber. Primary antibodies are listed in Supplementary Table S1, Supplementary Material 1. Following washing coverslips were incubated with secondary antibodies conjugated to proprietary oligonucleotide arms (Navenibodies) for 1 h. Incubation with specific enzymes enables the formation and amplification of a DNA circle in the spots of protein proximity. Fluorescent dots, generated by binding of fluorescently labelled probes, were visualized using an inverted confocal microscope (Leica TCS SP8 X, Leica Microsystems) with the HC PL APOCS2 63×/1.40 oil-immersion objective, zoom set at 2×. Images were analysed using LAS X software 3.1.1 (Leica Microsystems) and ImageJ.

### Statistical analysis

GraphPad Prism version 9.0.0 (GraphPad Software) was used to analyse data. Data obtained from IF or PLA assay were analysed by one-way analysis of variance (ANOVA) with Dunnett’s multiple comparison, and expressed as scatter plots with marked median. ns denotes not significant; * denotes *p* < 0.05; ** denotes *p* < 0.01; *** denotes *p* < 0.001; **** denotes *p* < 0.0001.

## Results

### Both melanoma cell lines MDA-MB-435S and RPMI-7951 display integrin αVβ5-positive, vinculin-negative structures in the cell centre

**Fig. 1:**
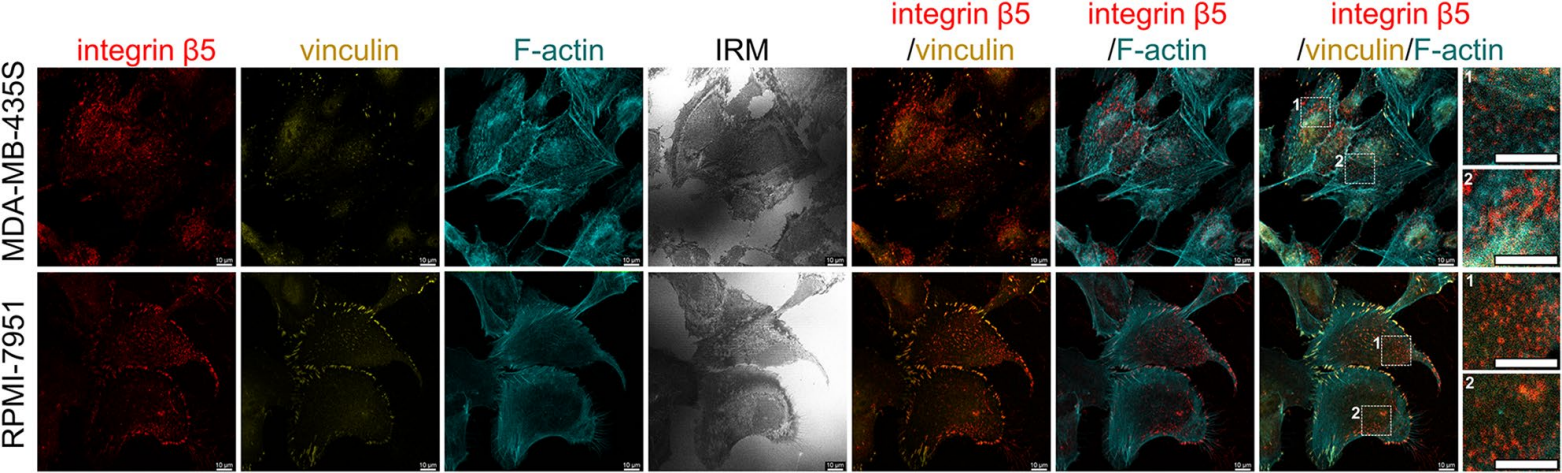
Melanoma cell lines MDA-MB-435S and RPMI-7951 contain integrin β5-positive, vinculin- and F-actin-negative structures in the cell centre. Forty-eight hours after seeding, cells were fixed with 4% PFA, and stained with anti-integrin β5 antibody followed by Alexa-Fluor 647-conjugated antibody (red) and anti-vinculin Alexa-Fluor 555-conjugated primary antibody (yellow). F-actin staining (cyan) was performed and IRM images were taken. Analysis was performed using TCS SP8 Leica. Scale bar = 10 μm

RAs primarily require integrin αVβ5, lack association with actin, and are devoid of markers of FAs, including vinculin [10, 11]. We have previously analysed IACs of two melanoma cell lines MDA-MB-435S and RPMI-7951, grown in long-term culture, using biochemical isolation and MS-based proteomics, and demonstrated that both cell lines preferentially use integrin αVβ5 for adhesion [30, 37]. These adhesome datasets were obtained from cells cultured for 48 h (MDA-MB-435S) or 72 h (RPMI-7951), on culture plates without prior ECM coating, to allow the cells to regulate their own environment by secreting ECM proteins. In these cultures, integrin αVβ5 was localized at the periphery in vinculin-positive FAs, but also in the central part of the cell in vinculin-negative structures, which were not associated with actin fibers (Fig. 1). This suggests that both melanoma cell lines, MDA-MB-435S and RPMI-7951, form RAs.

### Composition of IACs not associated with actin in melanoma cell lines MDA-MB-435S and RPMI-7951

Since RAs are formed independently of the actin cytoskeleton both cell lines were exposed to CytoD, an inhibitor of actin polymerisation known to disrupt FAs, as previously described [10, 23]. To determine the optimal CytoD concentration for enriching RAs, cells were treated with increasing doses for 2 h and IF was employed to visualize F-actin, the FA marker vinculin, the RA component Numb and the nucleus [10]. CytoD disrupted actin and FAs in a dose-dependent manner. Numb remained unaffected and progressively clustered beneath the nucleus, in a pattern characteristic of RAs. The most pronounced effect was observed at the CytoD concentration of 20 µM, as described in [10] and was used for all subsequent experiments (See Supplementary Fig. S1, Supplementary Material 2).

Next, IACs were isolated using a previously optimized protocol [30, 37] from CytoD treated cells with enriched RAs (CytoD(+), i.e. RAs) and compared to IAC isolates from cells treated with solvent DMSO (CytoD(-), i.e. FAs and RAs). IAC isolation and LC-MS/MS analysis were performed from four biological replicates of MDA-MB-435S (See Supplementary Table S2.1., Supplementary Material 3) and RPMI-7951 (See Supplementary Table S2.2., Supplementary Material 3) cells, and spectral counts were used as a measure of protein abundance. MS analysis identified 746 proteins for MDA-MB-435 S and 655 proteins for RPMI-7951 cells.

Integrin subunits that appeared in CytoD(-) samples of both cell lines were αV and β5 (See Supplementary Table S2.1., S2.2., Supplementary Material 3), again confirming previously published data that both cell lines preferentially use integrin αVβ5 for adhesion [29, 30, 37]. The next most abundant integrin subunit in both cell lines was β1, known to pair with many different integrin α subunits including αV and α5 [48].

**Fig. 2:**
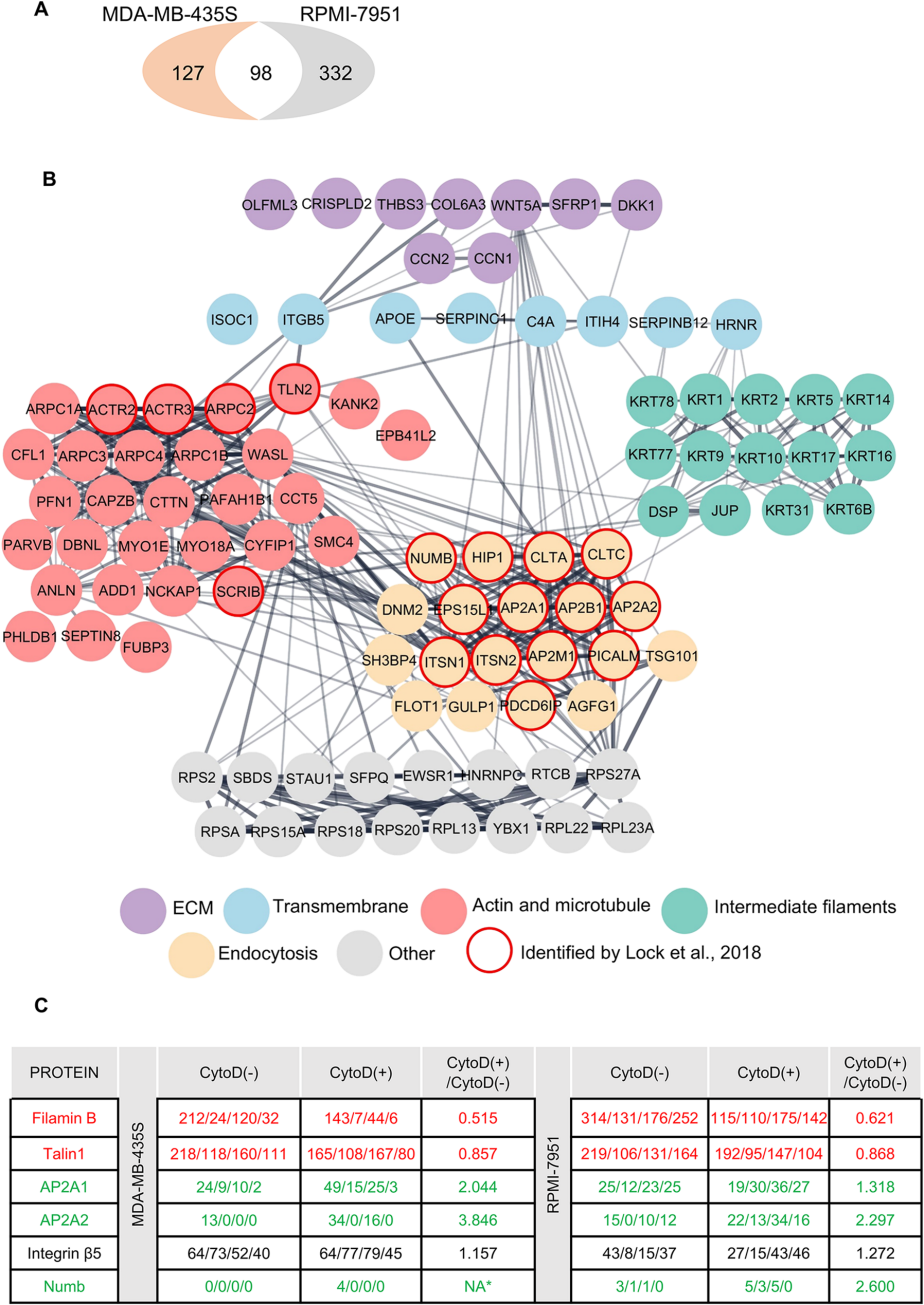
MS analysis of RAs isolated from MDA-MB-435S and RPMI-7951 melanoma cell lines. **A** Venn diagram represents the number of RA proteins enriched only in MDA-MB-435S cells (orange) and the number of RA proteins enriched only in RPMI-7951 cells (grey). Enriched RA proteins found in both cell lines are shown in the intersected white area of the diagram. **B** PPI network identified by MS in RAs isolated from both MDA-MB-435S and RPMI-7951 cells. Identified proteins are labelled with gene symbols, arranged and coloured according to their functional group as indicated. **C** Excerpt from MS data (See Supplementary Table S2.1 and S2.2., Supplementary Material 3) showing the number of protein-specific spectra for FAs proteins (red), RAs proteins (green) and integrin β5 present in both FAs and RAs (black). Dataset consists of four replicas. CytoD(+)/CytoD(-) ratio shown

To determine the differences in IAC composition between CytoD(-) and CytoD(+) samples, in both cell lines, the method of Lock et al. [10] was employed. They defined proteins as being RA components if a greater than 2-fold increase in abundance was observed following CytoD treatment. This threshold was lowered to 1.1 since in our experiments these ratios for typical RA proteins were lower. Another reason for using a lower ratio was to enable the detection of proteins present in both FAs and RAs, such as talin2 and integrin β5, and whose ratios in the MDA-MB-435S and RPMI-7951 cell lines were 1.334 and 1.225, and 1.157 and 1.272, respectively (Supplementary Table S2.3., Supplementary Material 3). This narrowed the list of candidate proteins to 225 for MDA-MB-435S cells and 430 for RPMI-7951 cells (See Supplementary Table S2.1., S2.2., Supplementary Material 3), of which 98 were shared between the cell lines (Fig. 2A, See Supplementary Table S2.3., Supplementary Material 3) and became the focus of future analysis.

A constructed Protein-Protein Interaction (PPI) network of the 98 enriched proteins (Fig. 2B; See Supplementary Table S2.1, S2.2., Supplementary Material 3) included several ECM proteins including collagen type VI alpha 3 chain (COL6A3), cysteine-rich angiogenic inducer 61 (CYR61, i.e. CCN1), thrombospondin-3 (THBS3) and Wnt family member 5 A (WNT5A). Transmembrane proteins identified within the network included integrin αV (ITGAV) and β5 (ITGB5), apolipoptotein E (APOE), and hornerin (HRNR).

Actin and microtubule-related proteins identified within the PPI network (Fig. 2B) comprised actin-related proteins 2 and 3 (ACTR2, ACTR3), components of the Arp2/3 complex (ARPC1A, ARPC1B, ARPC2, ARPC3, ARPC4), myosin IE and myosin XVIIIA (MYO1E, MYO18A), cortactin (CTTN), anillin (ANLN), talin2 (TLN2), cofilin-1 (CFL1), septin-8 (SEPTIN8) and others. It has been shown that Arp2/3-mediated actin polymerization through cortactin [49] facilitates the transition of clathrin plaques into endocytic pits, underscoring a mechanistic role for actin in endocytosis at plaque peripheries [12].

There was also an increase in abundance of several keratins in both cell lines (Fig. 2B). Since keratins can originate from MS sample preparation contamination [50] and provide false positives, and thus far, no literature data link keratins to RAs, we did not evaluate their significance.

CytoD(+) samples in MDA-MB-435S and RPMI-7951 cells showed a higher abundance of clathrin adaptor proteins AP2A1, AP2A2, AP2B1 and AP2M1, clathrin light and heavy chains CLTA and CLTC, Numb, phosphatidylinositol-binding clathrin assembly protein (PICALM), PTB domain-containing engulfment adaptor protein 1 (GULP1), programmed cell death 6-interacting protein (PDCD6IP), SH3 domain-binding protein 4 (SH3BP4), Huntingtin-interacting protein 1 (HIP1), flotillin 1 (FLOT1), Arf-GAP domain and FG repeat-containing protein 1 (AGFG1), scaffold proteins in endocytosis intersectins 1 and 2 (ITSN1 and ITSN2), dynamin2 (DNM2) and epidermal growth factor receptor substrate 15-like 1 protein (EPS15L1) (Fig. 2B). It should be noted that, of the 98 proteins enriched upon CytoD treatment, 18 are part of the reticular adhesome determined by Lock et al. [10] (Fig. 2B (circled red); See Supplementary Table S2.4., Supplementary Material 3), and 13 of those are proteins involved in endocytosis, while the rest are actin-related. Stonin1, which has been identified as a marker for RAs [25] was identified with a low number of spectra only in MS samples of MDA-MB-435S, but not RPMI-7951 cells. This could be due to the fact that stonin1 exhibits variable expression across different human cell types [51].

MS analysis identified several ribosomal proteins (ribosomal protein large (RPL) and small (RPS)) and those related to RNA and protein synthesis such as ribosome maturation factor SBDS, RNA 2’,3’-cyclic phosphate and 5’-OH ligase RTCB and proline- and glutamine-rich splicing factor SFPQ. Previous studies have described the presence of ribosomes in FAs and their transport towards adhesion sites upon binding to the ECM [52]. Ribosomal protein SA (RPSA), first identified as a laminin-binding protein, has been shown to regulate ribosome biogenesis, cytoskeletal organisation, and nuclear functions [53, 54]. It has also been implicated in cell migration [55], tumor cell blebbing and extracellular vesicle shedding [56]. However, the role of these proteins in RA-enriched samples remains unknown.

Supplementary Material 3 (Supplementary Tables S2.1 and S2.2) contains quantitative MS data from four independent biological replicates per cell line, under both control and CytoD-treated conditions, along with the associated statistical evaluation. Given the quantitative nature of the MS approach, these results provide statistically significant and analytically robust evidence that constitutes the core basis of our analysis. However, to illustrate changes in protein abundance in the samples and to show the spectral count ratios used to identify proteins potentially present in RAs, Fig. 2C displays an excerpt of number of specific spectra identified for two FA marker proteins (filamin B and talin1), two proteins found in RAs (AP2A1 and AP2A2) and integrin β5, which is present in both FA and RA structures. A decrease in the abundance of filamin B and talin1 (in both cell lines) in the CytoD(+) compared to the CytoD(-) samples confirmed the loss of FAs. The sample enrichment with proteins of RAs was further demonstrated by increased ratio for AP2A1 and AP2A2 (in MDA-MB-435S), and for AP2A1 and AP2A2 and Numb (in RPMI-7951) in the CytoD(+) samples, which is in line with the literature [10, 11, 23]. Integrin β5 was detected in all samples (Supplementary Tables S2.1. and S2.2., Supplementary Material 3), consistent with its previously confirmed localization in both FAs and RAs [10, 11]. In conclusion, the results demonstrate that both MDA-MB-435S and RPMI-7951 cells form αVβ5 RAs.

Next, we isolated RAs and, following SDS-PAGE, analysed these proteins by WB on the same membrane (Supplementary Fig. S2, S6, S7, S8, S9 Supplementary Material 2). WB analysis also demonstrated differential protein abundance depending on whether the proteins are localized to FAs (filamin B, talin1), RAs (AP2 and Numb), both adhesion types (integrin β5), or not present/retained in IAC-enriched samples (negative control; cytosolic lactate dehydrogenase, LDH). Notably, WB robustly detected Numb, which showed enrichment following CytoD treatment in both cell lines. In RPMI-7951 cells, we additionally detected Dab2, a protein characteristic of RAs, which was not identified in the MS analysis. All antibodies used for WB demonstrated high specificity, an important point given that they were subsequently used for IF analysis.

### KANK2 is a component of RAs in MDA-MB-435S and RPMI-7951 cells

**Fig. 3:**
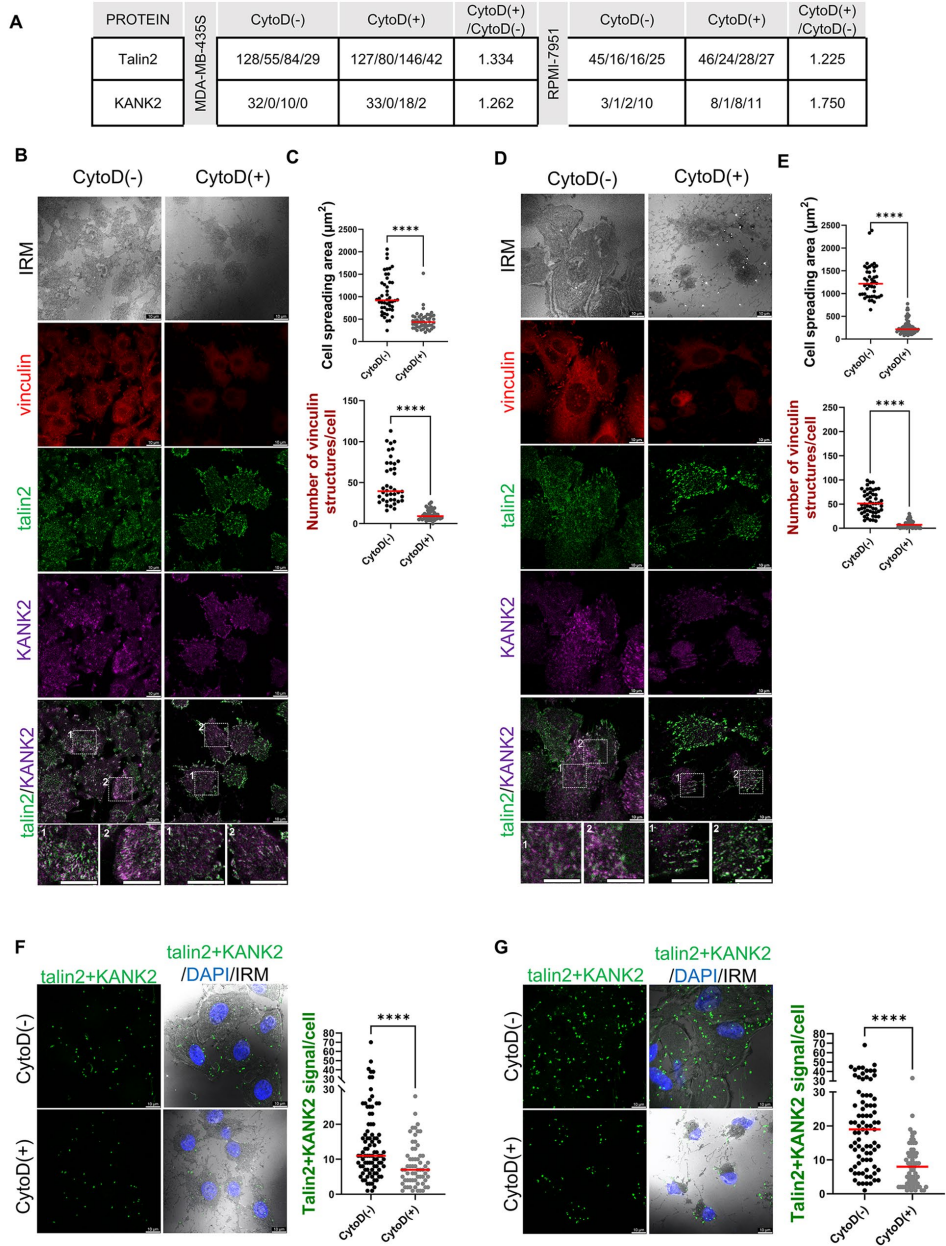
KANK2 colocalizes with talin2 in vinculin-negative structures in two melanoma cell lines MDA-MB-435S and RPMI-7951. **A** Excerpt from MS data (See Supplementary Table S2.1 and S2.2., Supplementary Material 3) showing the number of spectra specific for talin2 and KANK2 in MDA-MB-435S and RPMI-7951 cells. Dataset consists of four replicas. CytoD(+)/CytoD(-) ratio shown for both cell lines. **B**, **D** Talin2 and KANK2 are localized within RAs of both cell lines. Forty-eight hours after seeding, and 2 h after CytoD treatment, cells were methanol fixed, stained with anti-vinculin Alexa-Fluor 555-conjugated primary antibody (red), anti-talin2 antibody followed by Alexa-Fluor IgG2b 488-conjugated antibody (green) and anti-KANK2 antibody followed by Alexa-Fluor 647-conjugated antibody (magenta). IRM images were taken. Analysis was performed using TCS SP8 Leica. Scale bar = 10 μm. **C**, **E** Quantification of data presented in (**B**, **D**) respectively. Scatter plots (number of structures/cell or cell spreading area) with marked median represent measurements of > 40 cells. Data were analysed by one-way ANOVA with Dunnett’s multiple comparison. ns, not significant; * *P* < 0.05; ** *P* < 0.01; *** *P* < 0.001; **** *P* < 0.0001. **F**, **G** Talin2 and KANK2 are in close proximity in RAs of both cell lines. Forty-eight hours after seeding, and 2 h after CytoD treatment, RPMI-7951 cells were methanol fixed, and a PLA assay with anti-talin2 and anti-KANK2 primary antibody was performed. Generated fluorescent dots were visualized and IRM images were taken. Analysis was performed using TCS SP8 Leica. Scale bar = 10 μm. Quantification of data is presented in scatter plots with marked median (measurements of > 60 cells). Data were analysed by one-way ANOVA with Dunnett’s multiple comparison. ns, not significant; * *P* < 0.05; ** *P* < 0.01; *** *P* < 0.001; **** *P* < 0.0001

In addition to talin2, which is known to be present in FAs and RAs [10], MS data also showed an increased abundance of KANK2 following CytoD treatment in MDA-MB-435S and RPMI-7951 cells (Fig. 3A; See Supplementary Table S2.1, S2.2., S2.3., Supplementary Material 3). KANK2 is a known interactor of talin2 [29] and these results suggest that it may represent a previously unrecognized component of RAs. WB analysis also shows KANK2 enrichment in CytoD(+) samples from both MDA-MB-435S and RPMI-7951 cells suggesting that KANK2 is present in RAs (Supplementary Fig. S2, S8, S9, Supplementary Material 2).

IF analysis demonstrated that talin2, which we recently showed to colocalize with KANK2 in both FAs and FBs [29, 30], also colocalized with KANK2 following CytoD treatment, when FAs were disrupted (Fig. 3B, D). This observation was supported by quantification showing reduced cell spreading area and the loss of FA marker vinculin in both cell lines (Fig. 3C, E). In MDA-MB-435S cells, MS analysis of four independent biological replicates yielded CytoD(+)/CytoD(−) ratios of 1.334 for talin2 and 1.262 for KANK2 (Fig. 3A), and IF analysis showed that talin2- and KANK2-positive structures persisted after CytoD treatment (Fig. 3B). Similar results were obtained in RPMI-7951 cells, with CytoD(+)/CytoD(−) ratios of 1.225 for talin2 and 1.750 for KANK2 (Fig. 3A), and persistence of talin2- and KANK2-positive structures following CytoD treatment by IF (Fig. 3D), which most likely correspond to RAs.

The main objective of our study was to demonstrate that talin2 and KANK2 localize within the same adhesion structures. To address this, we employed PLA assay, which detects proteins located within approximately 40 nm of each other. PLA analysis of talin2 and KANK2 was performed and quantified in cells before and after CytoD treatment. Appropriate PLA controls are shown in Supplementary Fig. S3, Supplementary Material 2. Following CytoD-induced actin disruption, PLA signals between talin2 and KANK2 remained detectable in RAs (Fig. 3F, G). Quantification of PLA-positive signals in both cell lines revealed that actin disruption and the concomitant loss of FAs significantly reduced, but did not abolish, talin2-KANK2 signals (Fig. 3F, G). Overall, the persistence of talin2-KANK2 PLA signals after CytoD treatment supports their spatial association within RAs.

**Fig. 4:**
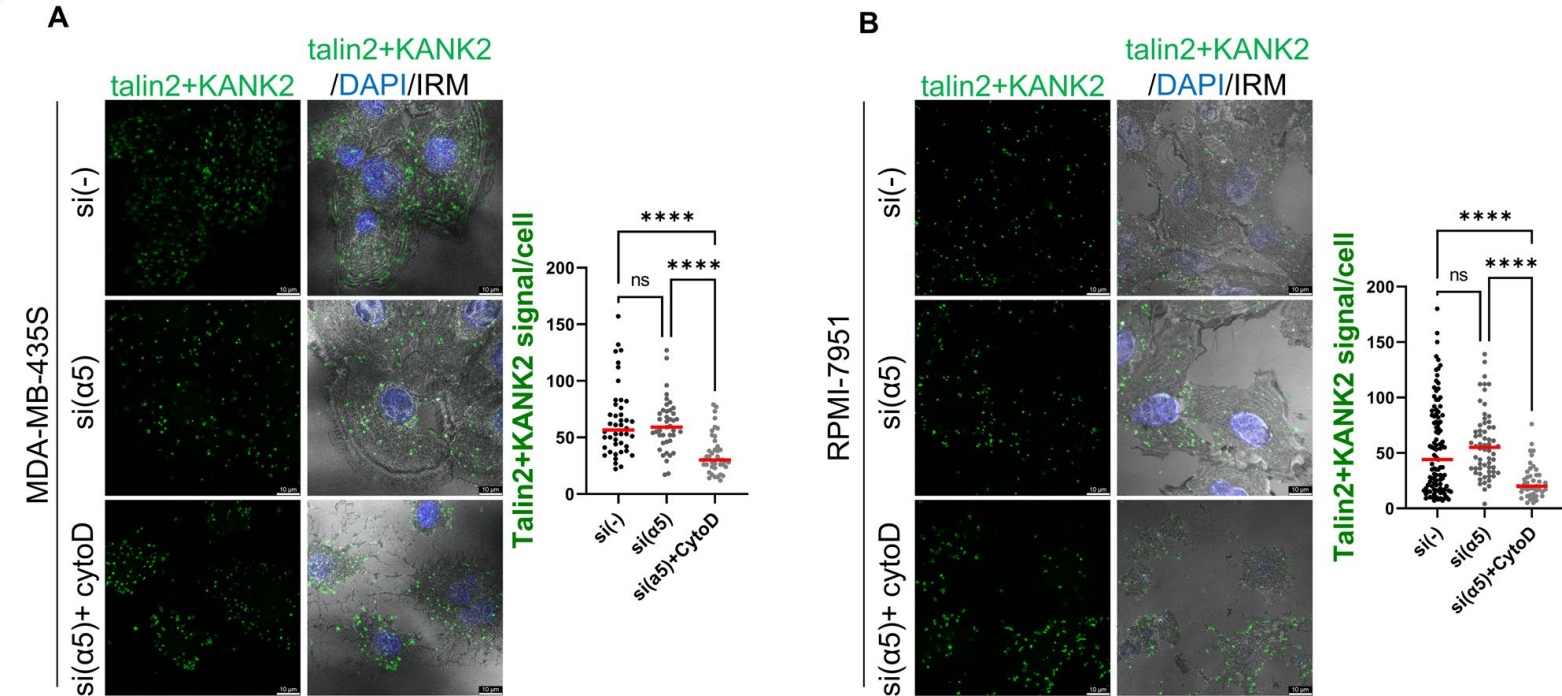
KANK2 localizes within RAs and is in close proximity to talin2 in melanoma cell lines MDA-MB-435S (**A**) and RPMI-7951 (**B**). Forty-eight hours after transfection with either control siRNA (si(-)) or integrin α5-specific siRNA (si(ITGA5)), followed by 2 h CytoD treatment, cells were methanol fixed, and a PLA assay with anti-talin2 and anti-KANK2 primary antibody was performed. Generated fluorescent dots were visualized and IRM images were taken. Analysis was performed using TCS SP8 Leica. Scale bar = 10 μm. Quantification of data is presented in scatter plots with marked median (measurements of > 40 cells). Data were analysed by one-way ANOVA with Dunnett’s multiple comparison. ns, not significant; * *P* < 0.05; ** *P* < 0.01; *** *P* < 0.001; **** *P* < 0.0001

The adhesome of MDA-MB-435S cells contained integrin β1 alongside αV and β5, but no spectra for integrin α5 were detected (see Supplementary Table 2.1., Supplementary Material 3), and integrin α5-positive structures characteristic of FBs were not observed (See Supplementary Fig. S4A, Supplementary Material 2). In contrast, the adhesome of RPMI-7951 cells contained αV, β5, and β1, as well as low levels of integrin α5 (See Supplementary Table S2.2., Supplementary Material 3) and these cells formed integrin α5-positive structures characteristic of FBs (See Supplementary Fig. S4A, Supplementary Material 2). Our recent data show that RPMI-7951 cells, when cultured on uncoated surfaces, formed elongated, integrin α5-positive structures characteristic of FBs containing talin2 and KANK2 [30]. To further confirm that KANK2 and talin2 are indeed components of RAs, both cell lines were transfected with integrin subunit α5-specific siRNA, treated with CytoD (to disrupt FAs) and PLA was performed, with talin2-KANK2 PLA-positive signals subsequently quantified. The efficiency of integrin α5 knockdown in RPMI-7951 cells is shown in Supplementary Fig. S4B, Supplementary Material 2. In MDA-MB-435S cells, which do not form FBs, integrin α5 knockdown had no effect on the number of talin2-KANK2 positive signals whereas CytoD treatment caused a reduction in their number, although signals remained detectable. Similarly, in RPMI-7951 cells, integrin α5 knockdown did not significantly affect talin2-KANK2 signals, whereas CytoD treatment caused a significant reduction, although the signals remained detectable (Fig. 4A, B). Taken together, these results identify KANK2 as a previously unrecognized component of RAs.

### The effect of talin2 or KANK2 knockdown on RAs abundance in MDA-MB-435S and RPMI-7951 cells

In MDA-MB-435S cells, talin2 and KANK2 within integrin αVβ5 FAs functionally interact to regulate microtubule dynamics and cell migration. More specifically, knockdown of either talin2 or KANK2 mimics the effect of integrin αV or β5 subunit knockdown i.e. reduces cell migration [29]. However, in RPMI-7951 cells, talin2 and KANK2 colocalize in FAs and FBs, and their knockdown has differential effect. Talin2 knockdown mimicked the effect of αV or β5 integrin knockdown i.e. reduced cell migration, while KANK2 knockdown mimicked the effect of integrin α5 knockdown i.e. enhanced cell migration [30]. These findings suggest that the localization of KANK2 to FAs or FBs differentially affects cell migration [29, 30].

**Fig. 5:**
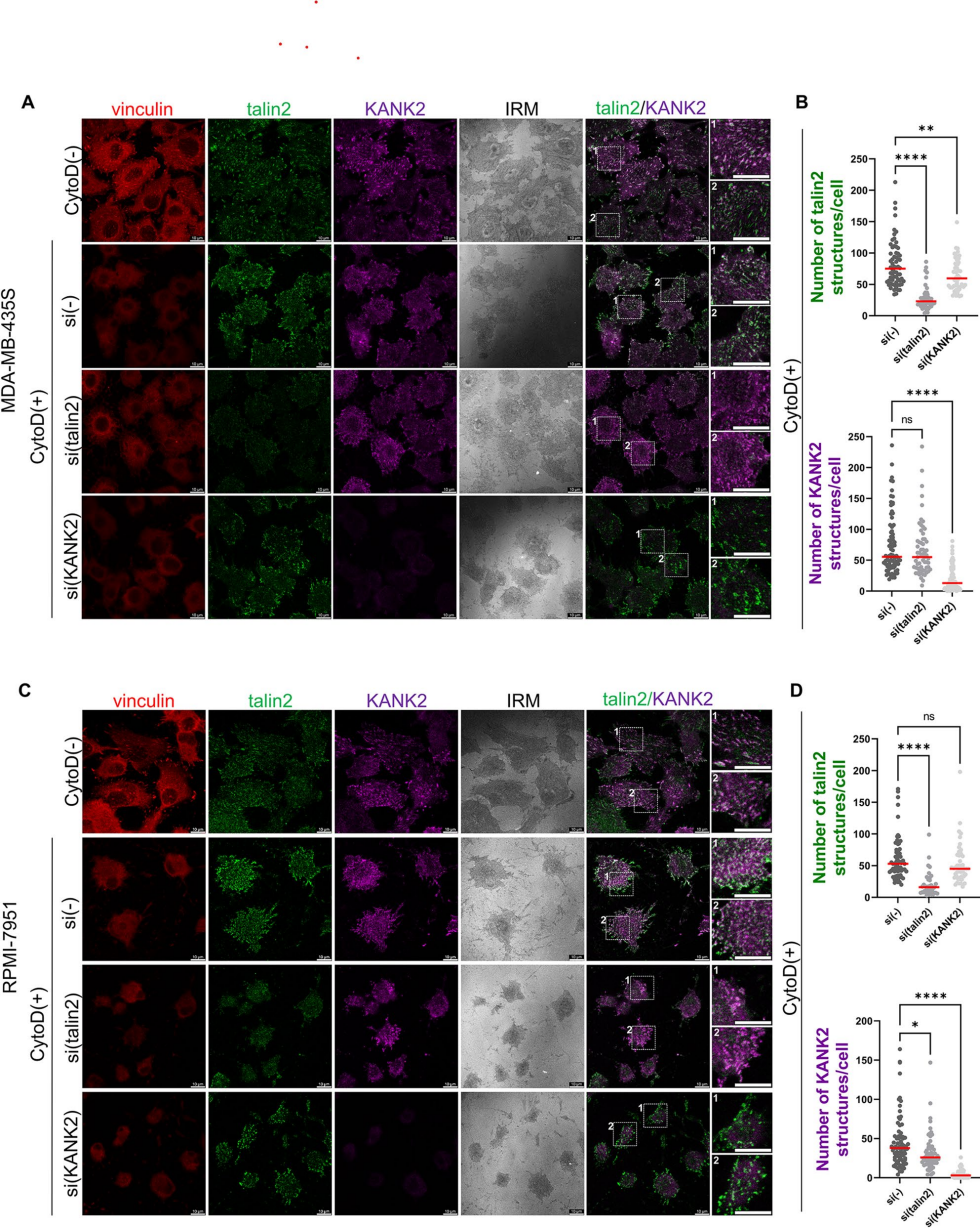
Neither talin2 nor KANK2 are essential for RA maintenance. Forty-eight hours after transfection with either control siRNA (si(-)), talin2-specific (si(talin2)) or KANK2-specific (si(KANK2)) siRNA, followed by 2 h CytoD treatment, MDA-MB-435S (**A**) and RPMI-7951 (**C**) cells were methanol fixed, and stained with anti-vinculin Alexa-Fluor 555-conjugated primary antibody (red), anti-talin2 antibody followed by Alexa-Fluor IgG2b 488-conjugated antibody (green) and anti-KANK2 antibody followed by Alexa-Fluor 647-conjugated antibody (magenta). IRM images were taken. Analysis was performed using TCS SP8 Leica. Scale bar = 10 μm. (**B**, **D**) Quantification of data presented in (**A**, **C**) respectively. Scatter plots represent measurements of > 40 cells. Data were analysed by one-way ANOVA with Dunnett’s multiple comparison. ns, not significant; * *P* < 0.05; ** *P* < 0.01; *** *P* < 0.001; **** *P* < 0.0001

**Fig. 6:**
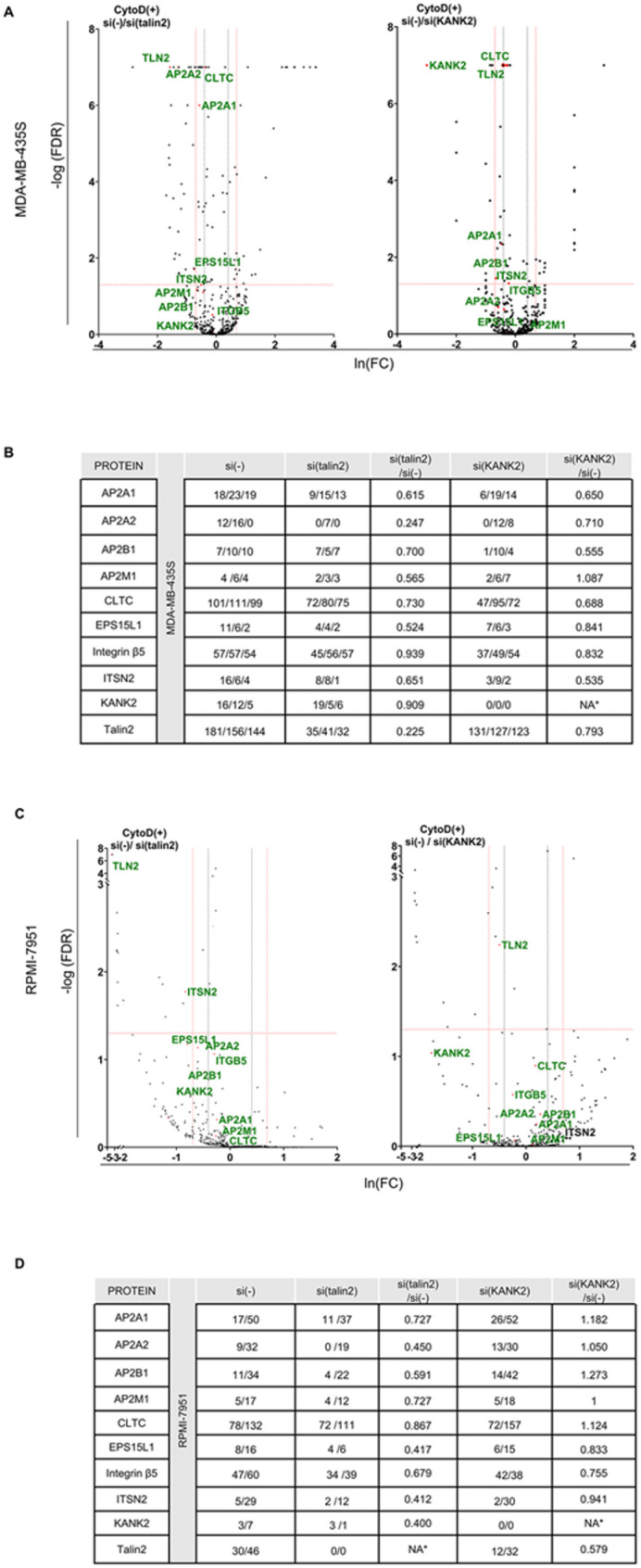
Talin2 and KANK2 knockdown differentially alters RA component abundance in MDA-MB-435S versus RPMI-7951 cells. Volcano plot analysis of proteins detected in RAs isolated from (**A**) MDA-MB-435S and (**C**) RPMI-7951 cells transiently transfected with control siRNA (si(-)) versus talin2-specific siRNA (si(talin2)) or KANK2-specific siRNA (si(KANK2)). RA proteins are visualized as volcano plot after the analysis with QSpec/QProt to generate -Log (FDR) and fold change values. Cut off values of − Log (FDR) ≥ 0.05 (red horizontal dotted line) corresponding to FDR ≤ 0.05; and fold change ≥ 1.5 (black vertical dotted line) or 2 (red vertical dotted line) were used. Each dot on the plot represents one protein. Proteins with significantly different abundance between RAs of si(talin2) or si(KANK2) and si(-) cells and of interest for this paper were marked with their gene name (green). Upper left quadrant – proteins detected with lower levels of spectra in si(talin2), upper right quadrant – proteins detected with higher levels of spectra in si(talin2) or si(KANK2). (**B**, **D**) Excerpt from MS data (See Supplementary Table S3.1 and S3.2., Supplementary Material 3) showing number of specific spectra for proteins presented in volcano plots (**A**, **C**) upon talin2 or KANK2 knockdown. Dataset consists of three replicas for MDA-MB-435S cells (**B**) and two replicas for RPMI-7951 cells (**D**). si(-)/si(talin2) or si(KANK2) ratio shown

To investigate how talin2 or KANK2 knockdown affects their mutual localization and/or abundance in RAs, each protein was knocked down in MDA-MB-435S and RPMI-7951 cells, and cells were then exposed to CytoD. Successful knockdown was confirmed in both cell lines using three independent methods: (i) MS analysis, performed in three biological replicates for MDA-MB-435S cells and two for RPMI-7951 cells (Fig. 6; Supplementary Tables S3.1–S3.2.2, Supplementary Material 3), (ii) WB analysis of isolated IACs following talin2 or KANK2 knockdown in both cell lines (representative images of two or more WBs; Supplementary Fig. S5, S11, S12 Supplementary Material 2) and (iii) protein localization, analysed by IF and quantification of talin2 and KANK2 positive structures after talin2 or KANK2 knockdown, with the loss of vinculin signal confirming the effectiveness of the CytoD treatment (Fig. 5). After verifying the efficiency of the knockdown with these approaches, the same methods were subsequently used to assess potential changes in protein overall abundance. In MDA-MB-435S cells, talin2 knockdown was highly effective (MS, WB, IF), resulting in a slight reduction in KANK2 abundance by MS and a strong reduction in WB analysis. KANK2 knockdown was also effective (MS, WB, IF), and markedly reduced talin2 levels in MS and WB. In RPMI-7951 cells, talin2 knockdown was highly effective in MS, WB and IF, and led to decreased number of KANK2 spectra in MS (although spectral counts were low) and clearly reduced levels in WB. KANK2 knockdown in RPMI-7951 cells was successful as judged by MS, WB and IF; however, MS indicated decreased abundance, and WB analysis showed an increase in talin2 levels following KANK2 knockdown. Regarding IF quantification, which detects the number of positive structures rather than protein abundance, the results show that in MDA-MB-435 S cells talin2 is reduced upon KANK2 knockdown, but not vice versa. This is in accordance to our previously published data that talin2 knockdown disrupted the link between talin2 and KANK2 in FAs but did not affect KANK2 localization [29]. Conversely, in RPMI-7951 cells knockdown of talin2 reduces KANK2 but not vice versa. This is also in accordance to our recently published data showing that KANK2 knockdown does not affect talin2 in β5 adhesions [30]. A major challenge in these analyses is that talin2 and KANK2 are present in all three types of adhesions, FAs, FBs, and RAs, so even a small fraction of adhesions that are not RAs (e.g., due to incomplete CytoD treatment) can affect the interpretation of data. Therefore, we conclude that knockdown of talin2 and KANK2 likely influences each other. Nevertheless, our primary goal was to assess how these knockdowns impact RA abundance, and in subsequent experiments we focused on spectral counts of proteins specifically characteristic of RAs. MS results (see Supplementary Table S3.1, S3.1.1, S3.1.2, S3.2, S3.2.1, S3.2.2., Supplementary Material 3) were processed, analysed as described above, and visualized using volcano plots. A significant change in the abundance of characteristic RA proteins was observed following talin2 knockdown in both cell lines, and after KANK2 knockdown in MDA-MB-435S, but not in the RPMI-7951 cell line (Fig. 6). In MDA-MB-435S cells, there was a significant reduction in the amount of several RA components such as AP2A1, AP2A2, EPS1L15, and ITSN2. KANK2 knockdown in MDA-MB-435 S cells also led to a decrease in the level of several RA components such as CTLC proteins, AP2A1, AP2B1, and ITSN2 (Fig. 6A, B). In RPMI-7951 cells, knockdown of talin2 similarly led to a decrease in the level of RA components AP2A1, AP2A2, AP2B1, CTLC, EPS1L15 and ITSN2 (Fig. 6C, D). These results are in line with observed changes in FAs of MDA-MB-435S after either talin2 or KANK2 knockdown and RPMI-7951 cells after talin2 knockdown. It is likely that FAs become less dynamic and their size increases, but not their number, indicating a possible cause for the retention of FA proteins [29, 30], which might lead to a reduction of RAs. However, whether this is indeed the underlying mechanism remains unclear, and further studies will be required to determine whether these processes are causally linked.

KANK2 knockdown did not have the same effect as talin2 knockdown in RPMI-7951 cells. The level of the RA components AP2A1, AP2A2, AP2B1, CTLC, EPS1L15 and ITSN2 did not change (Fig. 6B). This is in line to what happens with integrin αVβ5 FAs after KANK2 knockdown. Namely, KANK2 knockdown in RPMI-7951 did not affect integrin β5-positive adhesions at all [30].

## Discussion

Here, we report the composition of RAs in two melanoma cell lines, MDA-MB-435S and RPMI-7951, and compare the identified components with previously published data. The findings confirm previously obtained data by Lock et al. [10] that RAs contain talin2. Additionally, KANK2 is shown to be a novel component of RAs. Given that we have extensively studied the role and localization of talin2 and KANK2 in FAs and FBs of these two cell lines [29, 30], we further analysed how the knockdown of each protein affects RAs. Our results demonstrate that talin2 and KANK2 are not essential for the formation of RAs; however, their knockdown influences RA component abundance, and primarily mirrors the roles of talin2 and KANK2 in αVβ5 FAs. This highlights the importance of adhesion crosstalk in cellular function.

The analysis of IACs in the melanoma cell lines MDA-MB-435S and RPMI-7951 demonstrated that both cell lines in long-term culture preferentially use integrin αVβ5 for adhesion, forming FAs [29, 30] and RAs (this work) that contain talin2 and KANK2. It was also found that, unlike MDA-MB-435S cells, RPMI-7951 cells form FBs that also contain talin2 and KANK2 [30]. Here RAs were analysed in both cell lines and it was demonstrated that clathrin and endocytic adaptor proteins are enriched in RA isolates as previously reported by others [10, 11, 23]. In the analysis of RA proteins, the same logic as Lock and colleagues [10] was followed; i.e. the proteins we considered to be potential RA proteins were those maintained or enriched in the sample during RA isolation. In doing so, the presence of most of the previously identified proteins was confirmed in the melanoma cells [10]. Stonin1, which has been identified as a specific adaptor for the endocytosis of NG2 and as an important factor for FA dynamics and cell migration [57], and recently shown to be as a marker for RAs [25], was only detected in RA isolates of MDA-MB-435S, with a low number of spectra. In RPMI-7951 RA isolates, stonin1 was not detected, nor was it detected in RPMI-7951 IAC isolates [30]. This could be a consequence of the protein not being expressed in abundance in the melanoma cells. Therefore, in subsequent analyses of RA levels, endocytic adaptors such as AP2 were used as markers of RAs.

Previous research identified integrin αVβ5 as the main receptor in RAs; however, it is still not clear whether β3 and β1 can also contribute to their formation [10, 11]. MS analysis of isolated IACs after disruption of actin cytoskeleton using CytoD confirmed that αVβ5 integrin preferentially forms RAs in the two melanoma cell lines. Although a small number of spectra were found for several other integrin subunits, primarily β1, the α subunits did not overlap between the two cell lines. In RPMI-7951 cells, but not in MDA-MB-435S cells, MS analysis identified integrin α5, which is in line with our own results that these cells form FBs [30] and can be found in RAs isolates because FBs are less dependent on actin cytoskeleton.

Talin2 was found to be unnecessary for RA maintenance, which indicates that talin2 is not an activator of integrins in RA. Potential activators of integrin β5 in RA include the proteins Dab2, Numb, and ARH, which have been shown to interact directly with integrin β5 [58], and whose binding affinity for integrin β5 is several times higher than that of talin1 [23].

The key finding here is that KANK2 is a component of RAs in both melanoma cell lines. Not only was KANK2 enriched in samples of isolated RAs from both cell lines, those that form FBs and those that do not, but PLA analysis after integrin α5 knockdown (to deplete FBs) and CytoD treatment (to deplete FAs) also revealed close proximity between talin2 and KANK2. Therefore, given that talin2 and KANK2 were found in three different types of adhesions, this suggests that they may play a role in the interconversion. The fate of FAs and RAs has been suggested to be mainly connected via their shared use of αVβ5 integrins. Manipulations that inhibited one of these structures appeared to favour the other [10, 25] presumably by enlarging the available αVβ5 integrin pool [25]. Our results support these data. Namely, in MDA-MB-435S cell line talin2 from integrin αVβ5 FAs and KANK2 from CMSC have been implicated in maintaining actin-MT crosstalk. Therefore, knockdown of either of these proteins alters integrin αVβ5 FAs dynamics, resulting in enlarged αVβ5 FAs along with reduced cell migration [29]. The enlarged αVβ5 FAs suggest increased levels of FA proteins. Therefore, it was not surprising that knockdown of either talin2 or KANK2 led to reduced abundance of RA components. In RPMI-7951 cells, which form three talin2 and KANK2 positive adhesions (FAs, FBs and RAs), talin2 knockdown also increased FA size (suggesting increased levels of FA proteins) and reduced cell migration [30], and again a reduction in RAs levels was observed. However, in the RPMI-7951 cell line, KANK2 knockdown did not affect integrin αVβ5 FAs. Therefore, the unaltered αVβ5 FAs likely explains why the abundance of RA components remains unchanged after KANK2 knockdown.

The MS-data analysis showed that in MDA-MB-435S cells (which lack FBs), talin2 knockdown did not affect KANK2 levels, although the characteristic RA proteins were reduced. In RPMI-7951 cells, the very low number of KANK2-specific spectra made it impossible to determine whether KANK2 levels change; however, a clear decrease in specific RA proteins was observed. However, we believe that these data do not imply that KANK2 is not a part of RAs. Still, it remains unclear whether talin2 and KANK2 directly interact within RAs and what functional role they might play. Conversely, in both melanoma cell lines, KANK2 knockdown reduced talin2 levels, regardless of the overall effect on RA-specific proteins. This finding suggests the talin2 dependence of KANK2 levels. Moreover, it is in line with the previously mentioned observation that RA formation is independent of both talin2, already observed by [10], and apparently, KANK2 (our own data).

Until recently, it was not clear how αVβ5 integrin physically transfers between FAs and RAs. It has been shown that RAs arise more frequently at sites where the FA marker paxillin was progressively replaced by stonin1. In addition, the conversions in the opposite direction also occur by the recruitment of paxillin onto pre-existing β5 integrin scaffolds. These data indicate that two integrin αVβ5-based adhesions, having distinct molecular compositions, are intimately linked and dynamically interconvertible [25]. FAs and RAs are inversely regulated complexes and the balance between these types of adhesions is influenced by phosphatidylinositols [10]. High levels of activated myosin light chain (p-MLC) also correlated with integrin αVβ5 localizing to FAs over RAs [23]. Decreased membrane tension together with formation of contractile stress fibers, promotes the assembly of FAs, reduces RAs, and decreases cancer cell migration [59]. In MDA-MB-435S cells talin2 knockdown enhanced stress fiber formation and reduced cell migration. In addition, talin2 knockdown increased the size of FAs indicating preference of αVβ5 for FAs over RAs [29]. Therefore, our results are consistent with this mechanism of inverse regulation of FA and RA. These conclusions are further supported by results in the RPMI-7951 cell line, in which only talin2 alters FAs and consequently alters RAs.

This comprehensive analysis of RAs in two melanoma cell lines expands on previous findings and highlights novel molecular components. The presence of talin2 in RAs is confirmed and KANK2 is identified as a novel RA-associated protein in both cell lines. While neither protein is essential for RA formation, their knockdown significantly alters RA abundance and composition, reflecting their broader roles in other adhesion types such as FAs and FBs. These findings emphasize the importance of adhesion crosstalk and the dynamic balance between different adhesion structures, which is influenced by integrin composition, cytoskeletal integrity, and membrane tension.

## Supplementary Information

Supplementary Material 1.

Supplementary Material 2.

Supplementary Material 3.

Supplementary Material 4.

## Data Availability

The datasets supporting the conclusions of this article are available in the ProteomeXchange Consortium via the PRIDE partner repository, dataset identifier PXD071922, [https://www.ebi.ac.uk/pride/archive/]. Other raw data supporting the conclusions of this article will be made available by the authors, without undue reservation, to any qualified researcher.

## Abbreviations

IAC: Integrin adhesion complex
FA: Focal adhesion
FB: Fibrillar adhesion
RA: Reticular adhesion
CytoD: Cytochalasin D
PLA: Proximity ligation assay
KANK KN: motif and ankyrin repeat domain containing protein
ECM: Extracellular matrix
AP2: Adaptor protein 2
DAB2: Disabled homolog 2
NUMB: Endocytic Adaptor Protein Numb
EPS15: Epidermal Growth Factor Receptor Pathway Substrate
ITSN: Intersectin (ITSN)
LDLRAP1: Low Density Lipoprotein Receptor Adaptor Protein 1
MT: Microtubule
CMSC: Cortical microtubule stabilizing complex
ATCC: American Type Culture Collection
DMEM: Dulbecco's Modified Eagle Medium
FBS: Fetal Bovine Serum
ITGA5: Integrin Subunit α5
ITGB5: Integrin Subunit β5
TLN2: Talin2
WB: Western blotting
MS: Mass spectrometry
IF: Immunofluorescence analysis
HEPES: Hydroxyethylpiperazine ethanesulfonic acid
DTBP: Dimethyl 3,3’-dithiobispropionimidate, Wang and Richard’s reagent
LC-MS: Liquid Chromatography-Mass Spectrometry
PRIDE: PRoteomics IDEntifications Database
PPI: network Protein–Protein Interaction Network
STRING: Search Tool for the Retrieval of Interacting Genes/Proteins
SDS-PAGE: sodium dodecyl sulfate–polyacrylamide gel electrophoresis
IRM: Interference reflection microscopy
SD: Standard Deviation
COL6A3: Collagen type VI alpha 3 chain
CCN1: Cysteine-rich angiogenic inducer 61 CYR61
THBS3: Thrombospondin-3
WNT5A: Wnt family member 5 A
APOE: Apolipoptotein E
HRNR: Hornerin
ITGAV: Integrin αV
ACTR2: Actin-related protein 2
ACTR3: Actin-related protein3
ARPC1A: Actin Related Protein 2/3 Complex Subunit 1 A
ARPC1B: Actin Related Protein 2/3 Complex Subunit 1B
ARPC2: Actin Related Protein 2/3 Complex Subunit 2
ARPC3: Actin Related Protein 2/3 Complex Subunit 3
ARPC4: Actin Related Protein 2/3 Complex Subunit 4
MYO1E: Myosin IE
MYO18A: Myosin XVIIIA
CTTN: Cortactin
ANLN: Anillin
CFL1: Cofilin-1
SEPTIN8: Septin-8
AP2A1: Adaptor Related Protein Complex 2 Subunit Alpha 1
AP2A2: Adaptor Related Protein Complex 2 Subunit Alpha 1
AP2B1: Adaptor Related Protein Complex 2 Subunit Beta 1
AP2M1: Adaptor Related Protein Complex 2 Subunit Mu 1
CLTA: Clathrin light chain
CLTC: Clathrin heavy chain
PICALM: Phosphatidylinositol-binding clathrin assembly protein
GULP1 PTB: domain-containing engulfment adaptor protein 1
PDCD6IP: Programmed cell death 6-interacting protein
SH3BP4: SH3 domain-binding protein 4
HIP1: Huntingtin-interacting protein 1
FLOT1: Flotillin 1
AGFG1: Arf-GAP domain and FG repeat-containing protein 1
ITSN1: Intersectin 1
ITSN2: Intersectin 2
DNM2: Dynamin2
EPS15L1: Epidermal growth factor receptor substrate 15-like 1 protein
RPL: Ribosomal protein large
RPS: Ribosomal protein small
SBDS: Ribosome maturation factor
RTCB: RNA 2’,3’-cyclic phosphate and 5’-OH ligase
SFPQ: Proline- and glutamine-rich splicing factor
RPSA: Ribosomal protein SA

## References

[1] Chastney MR, Conway JRW, Ivaska J. Integrin adhesion complexes. Curr Biol. 2021;31(10):R536–42. 34033786 10.1016/j.cub.2021.01.038

[2] Mishra YG, Manavathi B. Focal adhesion dynamics in cellular function and disease. Cell Signal. 2021;85:110046. 34004332 10.1016/j.cellsig.2021.110046

[3] Yamaguchi N, Knaut H. Focal adhesion-mediated cell anchoring and migration: from in vitro to in vivo. Development. 2022;149(10):dev200647. 10.1242/dev.200647 PMC9188754 35587444

[4] Conway JRW, Jacquemet G. Cell matrix adhesion in cell migration. Essays Biochem. 2019;63(5):535–51. 31444228 10.1042/EBC20190012

[5] Katz BZ, Zamir E, Bershadsky A, Kam Z, Yamada KM, Geiger B. Physical state of the extracellular matrix regulates the structure and molecular composition of cell-matrix adhesions. Mol Biol Cell. 2000;11(3):1047–60. 10712519 10.1091/mbc.11.3.1047 PMC14830

[6] Pankov R, Cukierman E, Katz BZ, Matsumoto K, Lin DC, Lin S, et al. Integrin dynamics and matrix assembly: tensin-dependent translocation of alpha(5)beta(1) integrins promotes early fibronectin fibrillogenesis. J Cell Biol. 2000;148(5):1075–90. 10704455 10.1083/jcb.148.5.1075 PMC2174533

[7] Clark K, Pankov R, Travis MA, Askari JA, Mould AP, Craig SE, et al. A specific alpha5beta1-integrin conformation promotes directional integrin translocation and fibronectin matrix formation. J Cell Sci. 2005;118(Pt 2):291–300. 15615773 10.1242/jcs.01623 PMC3329624

[8] Zamir E, Katz M, Posen Y, Erez N, Yamada KM, Katz BZ, et al. Dynamics and segregation of cell-matrix adhesions in cultured fibroblasts. Nat Cell Biol. 2000;2(4):191–6. 10783236 10.1038/35008607

[9] Lu J, Doyle AD, Shinsato Y, Wang S, Bodendorfer MA, Zheng M, et al. Basement Membrane Regulates Fibronectin Organization Using Sliding Focal Adhesions Driven by a Contractile Winch. Dev Cell. 2020;52(5):631–46. e4. 32004443 10.1016/j.devcel.2020.01.007 PMC8335633

[10] Lock JG, Jones MC, Askari JA, Gong X, Oddone A, Olofsson H, et al. Reticular adhesions are a distinct class of cell-matrix adhesions that mediate attachment during mitosis. Nat Cell Biol. 2018;20(11):1290–302. 30361699 10.1038/s41556-018-0220-2

[11] Zuidema A, Wang W, Kreft M, Te Molder L, Hoekman L, Bleijerveld OB, et al. Mechanisms of integrin alphaVbeta5 clustering in flat clathrin lattices. J Cell Sci. 2018;131(21):jcs221317. 10.1242/jcs.221317 30301780

[12] Leyton-Puig D, Isogai T, Argenzio E, van den Broek B, Klarenbeek J, Janssen H, et al. Flat clathrin lattices are dynamic actin-controlled hubs for clathrin-mediated endocytosis and signalling of specific receptors. Nat Commun. 2017;8:16068. 28703125 10.1038/ncomms16068 PMC5511353

[13] Alfonzo-Mendez MA, Sochacki KA, Strub MP, Taraska JW. Dual clathrin and integrin signaling systems regulate growth factor receptor activation. Nat Commun. 2022;13(1):905. 35173166 10.1038/s41467-022-28373-x PMC8850434

[14] Baschieri F, Dayot S, Elkhatib N, Ly N, Capmany A, Schauer K, et al. Frustrated endocytosis controls contractility-independent mechanotransduction at clathrin-coated structures. Nat Commun. 2018;9(1):3825. 30237420 10.1038/s41467-018-06367-y PMC6148028

[15] Baschieri F, Porshneva K, Montagnac G. Frustrated clathrin-mediated endocytosis - causes and possible functions. J Cell Sci. 2020;133(11):jcs240861. 10.1242/jcs.240861 32499318

[16] Bruna-Gauchoux J, Montagnac G. Constraints and frustration in the clathrin-dependent endocytosis pathway. C R Biol. 2022;345(2):43–56. 36847464 10.5802/crbiol.88

[17] De Deyne PG, O’Neill A, Resneck WG, Dmytrenko GM, Pumplin DW, Bloch RJ. The vitronectin receptor associates with clathrin-coated membrane domains via the cytoplasmic domain of its beta5 subunit. J Cell Sci. 1998;111(Pt 18):2729–40. 9718366 10.1242/jcs.111.18.2729

[18] Saffarian S, Cocucci E, Kirchhausen T. Distinct dynamics of endocytic clathrin-coated pits and coated plaques. PLoS Biol. 2009;7(9):e1000191. 19809571 10.1371/journal.pbio.1000191 PMC2731173

[19] Lampe M, Vassilopoulos S, Merrifield C. Clathrin coated pits, plaques and adhesion. J Struct Biol. 2016;196(1):48–56. 27431447 10.1016/j.jsb.2016.07.009

[20] Bucher D, Mukenhirn M, Sochacki KA, Saharuka V, Huck C, Zambarda C, et al. Focal adhesion-generated cues in extracellular matrix regulate cell migration by local induction of clathrin-coated plaques. bioRxiv. 2018. 10.1101/493114.

[21] Moulay G, Laine J, Lemaitre M, Nakamori M, Nishino I, Caillol G, et al. Alternative splicing of clathrin heavy chain contributes to the switch from coated pits to plaques. J Cell Biol. 2020;219(9):e201912061. 10.1083/jcb.201912061 PMC7480091 32642759

[22] Hakanpaa L, Abouelezz A, Lenaerts AS, Culfa S, Algie M, Barlund J, et al. Reticular adhesions are assembled at flat clathrin lattices and opposed by active integrin alpha5beta1. J Cell Biol. 2023;222(8):e202303107. 10.1083/jcb.202303107 PMC10225744 37233325

[23] Zuidema A, Wang W, Kreft M, Bleijerveld OB, Hoekman L, Aretz J, et al. Molecular determinants of alphaVbeta5 localization in flat clathrin lattices - role of alphaVbeta5 in cell adhesion and proliferation. J Cell Sci. 2022;135(11):jcs259465. 10.1242/jcs.259465 PMC9234671 35532004

[24] Lock JG, Baschieri F, Jones MC, Humphries JD, Montagnac G, Stromblad S, et al. Clathrin-containing adhesion complexes. J Cell Biol. 2019;218(7):2086–95. 31208994 10.1083/jcb.201811160 PMC6605790

[25] Lukas F, Matthaeus C, Lopez-Hernandez T, Lahmann I, Schultz N, Lehmann M, et al. Canonical and non-canonical integrin-based adhesions dynamically interconvert. Nat Commun. 2024;15(1):2093. 38453931 10.1038/s41467-024-46381-x PMC10920918

[26] Klapholz B, Brown NH. Talin - the master of integrin adhesions. J Cell Sci. 2017;130(15):2435–46. 28701514 10.1242/jcs.190991

[27] del Rio A, Perez-Jimenez R, Liu R, Roca-Cusachs P, Fernandez JM, Sheetz MP. Stretching single talin rod molecules activates vinculin binding. Science. 2009;323(5914):638–41. 19179532 10.1126/science.1162912 PMC9339221

[28] Goult BT, Brown NH, Schwartz MA. Talin in mechanotransduction and mechanomemory at a glance. J Cell Sci. 2021;134(20):jcs258749. 10.1242/jcs.258749 PMC8697387 34708856

[29] Loncaric M, Stojanovic N, Rac-Justament A, Coopmans K, Majhen D, Humphries JD, et al. Talin2 and KANK2 functionally interact to regulate microtubule dynamics, paclitaxel sensitivity and cell migration in the MDA-MB-435S melanoma cell line. Cell Mol Biol Lett. 2023;28(1):56. 37460977 10.1186/s11658-023-00473-6 PMC10353188

[30] Stojanović N, Rac A, Lončarić M, Tadijan A, Paradžik M, Acman M, et al. KANK2 at focal adhesion regulates their maintenance and dynamics, while at fibrillar adhesions it influences cell migration via microtubule-dependent mechanism. Cell Commun Signal. 2026;24:224. 10.1186/s12964-026-02771-w PMC13081611 41776630

[31] Praekelt U, Kopp PM, Rehm K, Linder S, Bate N, Patel B, et al. New isoform-specific monoclonal antibodies reveal different sub-cellular localisations for talin1 and talin2. Eur J Cell Biol. 2012;91(3):180–91. 22306379 10.1016/j.ejcb.2011.12.003 PMC3629562

[32] Atherton P, Konstantinou R, Neo SP, Wang E, Balloi E, Ptushkina M, et al. Tensin3 interaction with talin drives the formation of fibronectin-associated fibrillar adhesions. J Cell Biol. 2022;221(10):e202107022. 10.1083/jcb.202107022 PMC9462884 36074065

[33] Bouchet BP, Gough RE, Ammon YC, van de Willige D, Post H, Jacquemet G, et al. Talin-KANK1 interaction controls the recruitment of cortical microtubule stabilizing complexes to focal adhesions. Elife. 2016;5:e18124. 10.7554/eLife.18124 PMC4995097 27410476

[34] Sun Z, Tseng HY, Tan S, Senger F, Kurzawa L, Dedden D, et al. Kank2 activates talin, reduces force transduction across integrins and induces central adhesion formation. Nat Cell Biol. 2016;18(9):941–53. 27548916 10.1038/ncb3402 PMC6053543

[35] Goult BT, Yan J, Schwartz MA. Talin as a mechanosensitive signaling hub. J Cell Biol. 2018;217(11):3776–84. 30254032 10.1083/jcb.201808061 PMC6219721

[36] Chen NP, Sun Z, Fassler R. The Kank family proteins in adhesion dynamics. Curr Opin Cell Biol. 2018;54:130–6. 29909279 10.1016/j.ceb.2018.05.015

[37] Paradzik M, Humphries JD, Stojanovic N, Nestic D, Majhen D, Dekanic A, et al. KANK2 Links alphaVbeta5 Focal Adhesions to Microtubules and Regulates Sensitivity to Microtubule Poisons and Cell Migration. Front Cell Dev Biol. 2020;8:125. 32195252 10.3389/fcell.2020.00125 PMC7063070

[38] Jones MC, Humphries JD, Byron A, Millon-Fremillon A, Robertson J, Paul NR, et al. Isolation of integrin-based adhesion complexes. Curr Protoc Cell Biol. 2015;66:9. 8 1–9 8 15. 10.1002/0471143030.cb0908s66 PMC4402726 25727331

[39] Robertson J, Jacquemet G, Byron A, Jones MC, Warwood S, Selley JN, et al. Defining the phospho-adhesome through the phosphoproteomic analysis of integrin signalling. Nat Commun. 2015;6:6265. 25677187 10.1038/ncomms7265 PMC4338609

[40] Horton ER, Byron A, Askari JA, Ng DHJ, Millon-Fremillon A, Robertson J, et al. Definition of a consensus integrin adhesome and its dynamics during adhesion complex assembly and disassembly. Nat Cell Biol. 2015;17(12):1577–87. 26479319 10.1038/ncb3257 PMC4663675

[41] Nesvizhskii AI, Keller A, Kolker E, Aebersold R. A statistical model for identifying proteins by tandem mass spectrometry. Anal Chem. 2003;75(17):4646–58. 14632076 10.1021/ac0341261

[42] Deutsch EW, Bandeira N, Perez-Riverol Y, Sharma V, Carver JJ, Mendoza L, et al. The ProteomeXchange consortium at 10 years: 2023 update. Nucleic Acids Res. 2023;51(D1):D1539–48. 36370099 10.1093/nar/gkac1040 PMC9825490

[43] Perez-Riverol Y, Bandla C, Kundu DJ, Kamatchinathan S, Bai J, Hewapathirana S, et al. The PRIDE database at 20 years: 2025 update. Nucleic Acids Res. 2025;53(D1):D543–53. 39494541 10.1093/nar/gkae1011 PMC11701690

[44] Doncheva NT, Morris JH, Gorodkin J, Jensen LJ. Cytoscape StringApp: Network Analysis and Visualization of Proteomics Data. J Proteome Res. 2019;18(2):623–32. 30450911 10.1021/acs.jproteome.8b00702 PMC6800166

[45] Shannon P, Markiel A, Ozier O, Baliga NS, Wang JT, Ramage D, et al. Cytoscape: a software environment for integrated models of biomolecular interaction networks. Genome Res. 2003;13(11):2498–504. 14597658 10.1101/gr.1239303 PMC403769

[46] Choi H, Fermin D, Nesvizhskii AI. Significance analysis of spectral count data in label-free shotgun proteomics. Mol Cell Proteom. 2008;7(12):2373–85. 10.1074/mcp.M800203-MCP200 PMC2596341 18644780

[47] Alam MS. Proximity Ligation Assay (PLA). Curr Protoc Immunol. 2018;123(1):e58. 30238640 10.1002/cpim.58 PMC6205916

[48] Arias-Mejias SM, Warda KY, Quattrocchi E, Alonso-Quinones H, Sominidi-Damodaran S, Meves A. The role of integrins in melanoma: a review. Int J Dermatol. 2020;59(5):525–34. 32157692 10.1111/ijd.14850 PMC7167356

[49] Uruno T, Liu J, Zhang P, Fan Y, Egile C, Li R, et al. Activation of Arp2/3 complex-mediated actin polymerization by cortactin. Nat Cell Biol. 2001;3(3):259–66. 11231575 10.1038/35060051

[50] Hodge K, Have ST, Hutton L, Lamond AI. Cleaning up the masses: exclusion lists to reduce contamination with HPLC-MS/MS. J Proteom. 2013;88:92–103. 10.1016/j.jprot.2013.02.023 PMC3714598 23501838

[51] Lukas F, Duchmann M, Maritzen T. Focal adhesions, reticular adhesions, flat clathrin lattices: what divides them, what unites them? Am J Physiol Cell Physiol. 2025;328(1):C288–302. 39652817 10.1152/ajpcell.00821.2024

[52] Simpson LJ, Reader JS, Tzima E. Mechanical forces and their effect on the ribosome and protein translation machinery. Cells. 2020;9(3):650. 10.3390/cells9030650 PMC7140433 32156009

[53] Mercurio AM. Laminin receptors: achieving specificity through cooperation. Trends Cell Biol. 1995;5(11):419–23. 14732046 10.1016/s0962-8924(00)89100-x

[54] DiGiacomo V, Meruelo D. Looking into laminin receptor: critical discussion regarding the non-integrin 37/67-kDa laminin receptor/RPSA protein. Biol Rev Camb Philos Soc. 2016;91(2):288–310. 25630983 10.1111/brv.12170 PMC5249262

[55] Lefebvre T, Rybarczyk P, Bretaudeau C, Vanlaeys A, Cousin R, Brassart-Pasco S, et al. TRPM7/RPSA Complex Regulates Pancreatic Cancer Cell Migration. Front Cell Dev Biol. 2020;8:549. 32733880 10.3389/fcell.2020.00549 PMC7360683

[56] Brassart B, Da Silva J, Donet M, Seurat E, Hague F, Terryn C, et al. Tumour cell blebbing and extracellular vesicle shedding: key role of matrikines and ribosomal protein SA. Br J Cancer. 2019;120(4):453–65. 30739912 10.1038/s41416-019-0382-0 PMC6461924

[57] Feutlinske F, Browarski M, Ku MC, Trnka P, Waiczies S, Niendorf T, et al. Stonin1 mediates endocytosis of the proteoglycan NG2 and regulates focal adhesion dynamics and cell motility. Nat Commun. 2015;6:8535. 26437238 10.1038/ncomms9535 PMC4600748

[58] Calderwood DA, Fujioka Y, de Pereda JM, Garcia-Alvarez B, Nakamoto T, Margolis B, et al. Integrin beta cytoplasmic domain interactions with phosphotyrosine-binding domains: a structural prototype for diversity in integrin signaling. Proc Natl Acad Sci U S A. 2003;100(5):2272–7. 12606711 10.1073/pnas.262791999 PMC151330

[59] Djakbarova U, Madraki Y, Chan ET, Wu T, Atreaga-Muniz V, Akatay AA et al. Tension-induced adhesion mode switching: the interplay between focal adhesions and clathrin-containing adhesion complexes. bioRxiv. 2024:2024.02.07.579324.

