# Supplementary material for: Regulation of reticular adhesions by KANK2 and talin2 in two melanoma cell lines
Source: Cell Commun Signal. 2026 Apr 24;24:338. doi: 10.1186/s12964-026-02904-1 (PMC13244953; doi:10.1186/s12964-026-02904-1)
Supplement: Supplementary file 1 — Supplementary Material 1. [file 12964_2026_2904_MOESM1_ESM.pdf]

Additional file 1

Regulation of reticular adhesions by KANK2 and talin2 in two melanoma cell lines

Anja Rac[1,* ORCID:0000-0001-8821-3059], Marija Lončarić[1,* ORCID:0000-0002-5343-0368], Nikolina Stojanović[1,*,# ORCID:0000-0002-7763-4154], Mahak Fatima[2 ORCID: 0000-0003-2780-0844], Mirna Rešetar[1], Dalibor Hršak[3 ORCID: 0000-0002-1462-7424], Jonathan D. Humphries[4 ORCID:0000-0002-8953-7079], Martin J. Humphries[2 ORCID:0000-0002-4331-6967], Andreja Ambriović-Ristov[1,# ORCID:0000-0001-7784-2466]

[1]Laboratory for Cell Biology and Signalling, Division of Molecular Biology, Ruđer Bošković Institute, Zagreb, Croatia; [2]Manchester Cell-Matrix Centre, Faculty of Biology, Medicine & Health, University of Manchester, Manchester, United Kingdom; [3]Laboratory for Computational Biology and Translational Medicine, Division of Electronics, Ruđer Bošković Institute, Zagreb, Croatia; [4]Department of Life Science, Manchester Metropolitan University, Manchester, United Kingdom

*equal contribution

[#]corresponding authors: Nikolina.Stojanovic@irb.hr, Andreja.Ambriovic.Ristov@irb.hr

Table S1

Table S1. List of used antibodies and dyes.

**WESTERN BLOT**					
Primary antibodies	***Ref. No.***	***Distributor***	***Monoclonal/polyclonal***	***Species***	***Dilution***
Anti-Filamin B	ab97457	Abcam, USA	Monoclonal	Rabbit	1:1000 in 5% milk
Anti-human talin1	MCA4770GA	Bio-Rad, USA	Monoclonal	Mouse	1:1000 in 5% milk
Anti-a-Adaptin 1/2 (C-8) AP-2	sc-17771	Santa Cruz Biotechnology, USA	Polyclonal	Mouse	1:1000 in 5% milk
Anti-Integrin β5	D24A5	Cell Signaling Technology, USA	Monoclonal	Mouse	1:1000 in 5% milk
Anti-Numb (C29G11)	#2756	Cell Signaling Technology, USA	Monoclonal	Rabbit	1:1000 in 5% milk
Anti-LDH	sc33781	Santa Cruz Biotechnology, USA	Polyclonal	Rabbit	1:400 in 5% milk
Anti-human talin2	MCA4771GA	Bio-Rad, USA	Monoclonal	Mouse	1:1000 in 5% milk
Anti-Dab2 (D7O9T)	#12906	Cell Signaling Technology, USA	Monoclonal	Rabbit	1:1000 in 5% milk
Anti-KANK2	HPA015643	Sigma-Aldrich, USA	Polyclonal	Rabbit	1:1000 in 5% milk
Anti-Integrin α5	MAB18642	Bio-techne, USA	Monoclonal	Mouse	1:500 in 5% milk
Secondary antibodies	***Ref. No.***	***Distributor***	***Monoclonal/ polyclonal***	***Species***	***Dilution***
Goat anti-rabbit IgG (H+L)	31466	Invitrogen, USA	Polyclonal	Goat	1:5000 in 5% milk
Goat anti-mouse IgG (H+L)	G21040	Invitrogen, USA	Polyclonal	Goat	1:10 000 in 5% milk
**IMMUNOFLUORESCENCE**					
Primary antibodies	***Ref. No.***	***Distributor***	***Monoclonal/ polyclonal***	***Species***	***Dilution***
Anti-Integrin β5	D24A5	Cell Signaling Technology, USA	Monoclonal	Mouse	1:600 in 5% BSA
Anti-vinculin	ab129002	Abcam, USA	Monoclonal	Rabbit	1:100 in 5% BSA
Anti-human talin2	MCA4771GA	Bio-Rad, USA	Monoclonal	Mouse	1:100 in 5% BSA
Anti-KANK2	HPA015643	Sigma-Aldrich, USA	Polyclonal	Rabbit	1:100 in 5% BSA
Anti-Integrin α5	NBP2-50146	Novus Biologicals, USA	Monoclonal	Mouse	1:500 in 5% BSA
Anti-Numb (C29G11)	#2756	Cell Signaling Technology, USA	Monoclonal	Rabbit	1:600 in 5% BSA
Recombinant Alexa Fluor® 647 Anti-Vinculin	ab196579	Abcam, UK	Monoclonal	Rabbit	1:200 in 5% BSA
Secondary antibodies	***Ref. No.***	***Distributor***	***Monoclonal/polyclonal***	***Species***	***Dilution***

Anti-Mouse IgG Alexa Fluor 546	A-11030	Invitrogen, USA	Polyclonal	Goat	1:1000 in 5% BSA
Anti-Mouse IgG Alexa Fluor 488	#4408	Cell Signaling Technology, USA		Goat	1:1000 in 5% BSA
Anti-Mouse IgG Alexa Fluor 405	A-31553	Invitrogen, USA	Polyclonal	Goat	1:250 in 5% BSA
Anti-Rabbit IgG Alexa Flour, 555	A-31572	Invitrogen, USA	Polyclonal	Donkey	1:1000 in 5% BSA
Anti-Rabbit IgG Alexa Fluor 647	#4414	Cell Signaling Technology, USA	Polyclonal	Goat	1:1000 in 5% BSA
Anti-Mouse IgG1 Alexa Fluor 555	A-21127	Invitrogen, USA	Polyclonal	Goat	1:1000 in 5% BSA
Anti-Mouse $IgG2_b$ Alexa Fluor 488	A-21141	Invitrogen, USA	Polyclonal	Goat	1:1000 in 5% BSA
Dyes	***Ref. No.***	***Distributor***			***Dilution***
Phalloidin, Alexa Fluor 488	P5282	Sigma Aldrich, USA			1:100 in 5% BSA
**PLA**					
Primary antibodies	***Ref. No.***	***Distributor***	***Monoclonal/polyclonal***	***Species***	***Dilution***
Anti-human talin2	MCA4771GA	Bio-Rad, USA	Monoclonal	Mouse	1:4000 in diluent*
Anti-KANK2	HPA015643	Sigma-Aldrich, USA	Polyclonal	Rabbit	1:4000 in diluent*
Secondary antibodies	***Ref. No.***	***Distributor***	***Monoclonal/ polyclonal***	***Species***	***Dilution***
Navenibody M1 (40X)	NB.1.100.06	Navinci Diagnostics AB, Sweden		Mouse	1:40 in diluent*
Navenibody R2 (40X)	NB.1.100.07	Navinci Diagnostics AB, Sweden		Rabbit	1:40 in diluent*

** part of NaveniFlexTM Cell MR kit*
