# Supplementary material for: Regulation of reticular adhesions by KANK2 and talin2 in two melanoma cell lines
Source: Cell Commun Signal. 2026 Apr 24;24:338. doi: 10.1186/s12964-026-02904-1 (PMC13244953; doi:10.1186/s12964-026-02904-1)
Supplement: Supplementary file 2 — Supplementary Material 2. [file 12964_2026_2904_MOESM2_ESM.pdf]

Supplementary Material 2

Regulation of reticular adhesions by KANK2 and talin2 in two melanoma cell lines

Anja Rac[1,* ORCID:0000-0001-8821-3059], Marija Lončarić[1,* ORCID:0000-0002-5343-0368], Nikolina Stojanović[1,*,# ORCID:0000-0002-7763-4154], Mahak Fatima[2 ORCID: 0000-0003-2780-0844], Mirna Rešetar[1], Dalibor Hršak[3 ORCID: 0000-0002-1462-7424], Jonathan D. Humphries[4 ORCID:0000-0002-8953-7079], Martin J. Humphries[2 ORCID:0000-0002-4331-6967], Andreja Ambriović-Ristov[1,# ORCID:0000-0001-7784-2466]

[1]Laboratory for Cell Biology and Signalling, Division of Molecular Biology, Ruđer Bošković Institute, Zagreb, Croatia; [2]Manchester Cell-Matrix Centre, Faculty of Biology, Medicine & Health, University of Manchester, Manchester, United Kingdom; [3]Laboratory for Computational Biology and Translational Medicine, Division of Electronics, Ruđer Bošković Institute, Zagreb, Croatia; [4]Department of Life Science, Manchester Metropolitan University, Manchester, United Kingdom

*equal contribution

[#]corresponding authors: Nikolina.Stojanovic@irb.hr, Andreja.Ambriovic.Ristov@irb.hr

Supplementary Methods

SDS-PAGE and Western Blotting

Total cell lysates were obtained from 3.5 cm Petri dishes in 200 µL RIPA buffer supplemented with protease inhibitor cocktail (ThermoFisher). Samples for SDS-PAGE were collected by scraping. Samples containing an equal amount of protein were mixed in 6× Laemmli loading buffer (375 mM Tris-HCl (pH 6.8), 30% (w/v) glycerol, 12% (w/v) SDS, 0.02% (w/v) bromophenol blue, 12% (v/v) 2-mercaptoethanol) to reach a final 1× concentration, sonicated and heated for 5 minutes at 96°C. Isolated IACs were prepared for SDS-PAGE by solubilization in 2× Laemmli loading buffer and heating for 20 minutes at 70°C while shaking (1000 rpm). All samples were loaded onto pre-casted gradient gel (4 – 15% Mini-PROTEAN TGX) (Bio-Rad), separated by SDS-PAGE and semi-dry transferred to nitrocellulose (Bio-Rad). The membrane was blocked in 5% (w/v) non-fat dry milk or 5% (w/v) bovine serum albumin (BSA, Carl Roth), and incubated overnight with the appropriate antibodies, followed by incubation with horseradish peroxidase coupled secondary antibody. The primary and secondary antibodies are listed in Supplementary Table S1, Supplementary Material 1. Detection was performed using chemiluminescence (PerkinElmer) and documented with Uvitec Alliance Q9 mini (BioSPX b.v.). Blots were quantified using ImageJ.

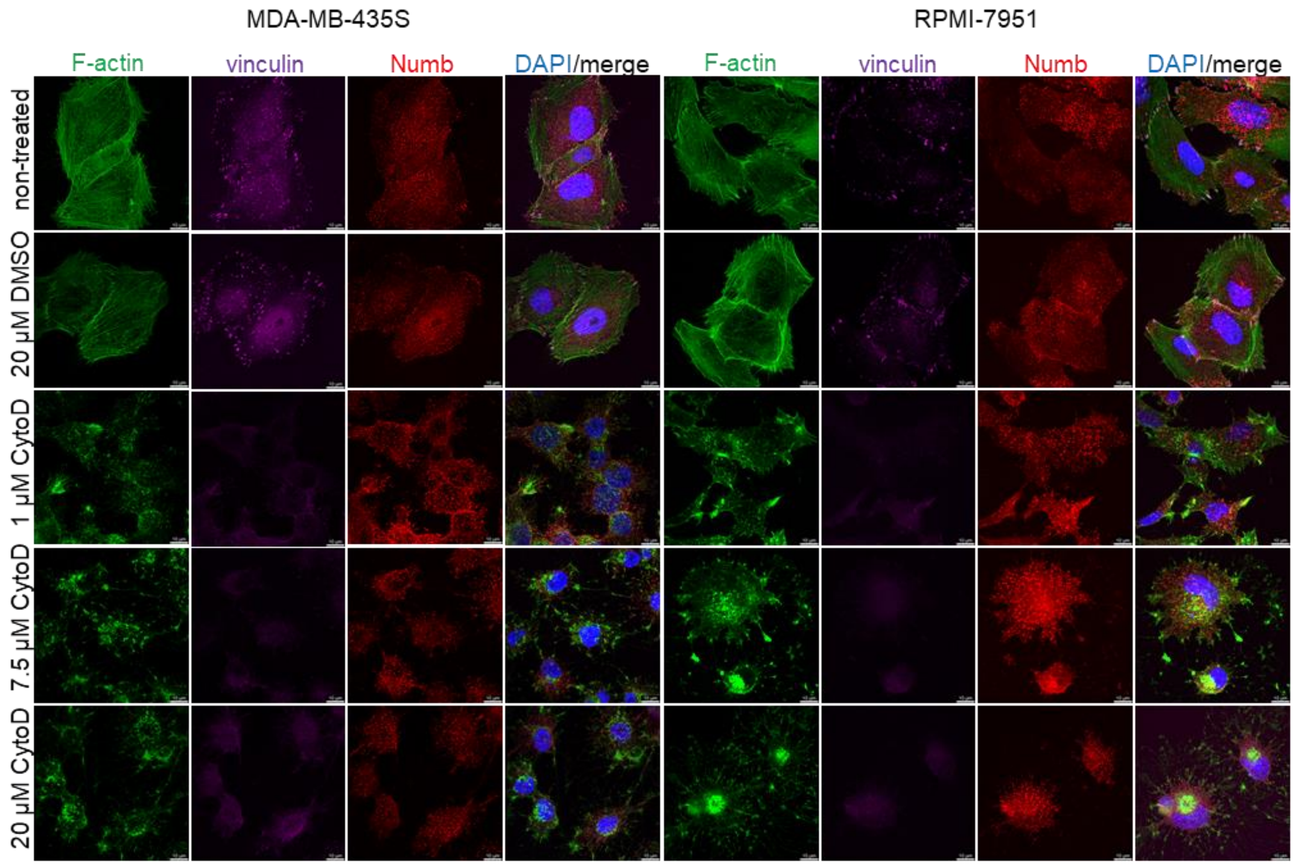

Supplementary Fig. S1 Optimization of CytoD concentration for enriching RAs in MDA-MB-435S and RPMI-7951 cells. Forty-eight hours upon seeding on coverslips, cells were treated with different concentrations of CytoD, fixed with PFA, permeabilised and stained with anti-vinculin and anti-Numb, followed by Alexa-Fluor 647-conjugated antibody (magenta) or Alexa-Fluor 546-conjugated antibody (red), respectively. F-actin staining (shown in green) was performed and IRM images were taken. Controls included non-treated cells and cells treated with 20 µM DMSO, a cytoD solvent. Analysis was performed using TCS SP8 Leica. Scale bar = 10 µm.

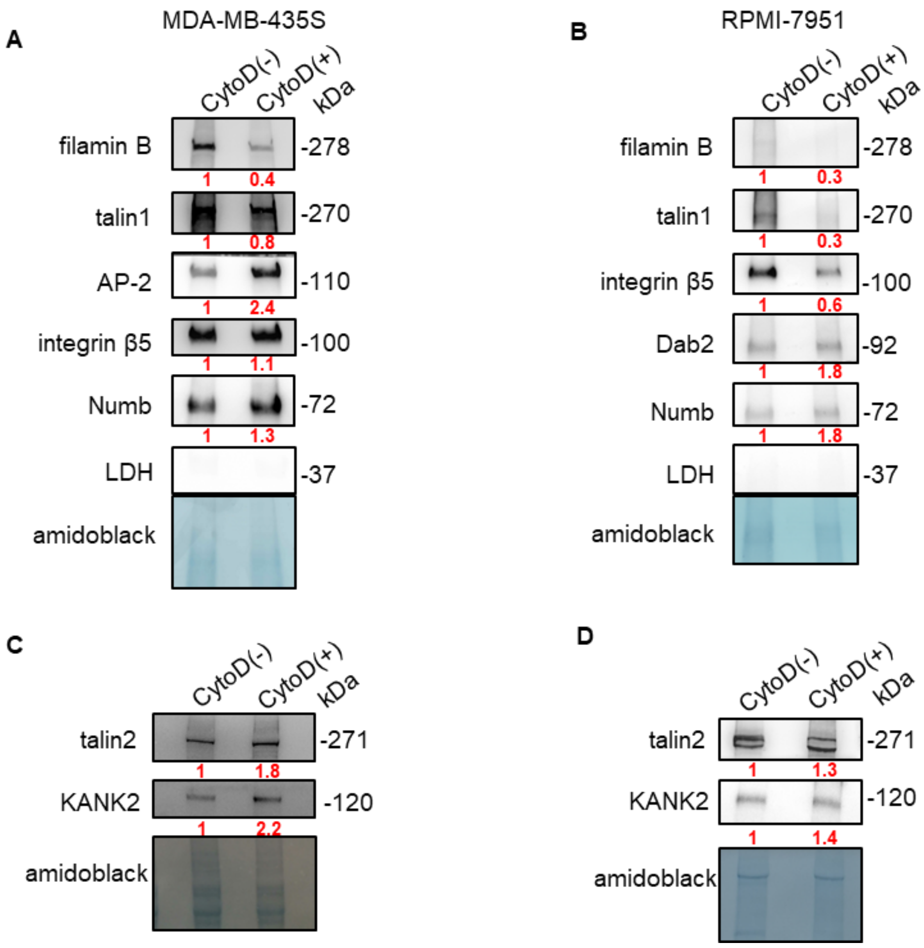

Supplementary Fig. S2 Validation of MS data with primary antibodies used in IF. (A, B) WB analysis of IAC proteins in MDA-MB-435S (A, C) and RPMI-7951 cells (B, D) in non-treated (CytoD(-)) and CytoD-treated (CytoD(+)) samples. Two hours upon CytoD treatment, IACs were isolated and WB analysis was performed. Amidoblack staining of the membrane was used as a loading control. The results presented are representative of at least two independent experiments yielding similar results.

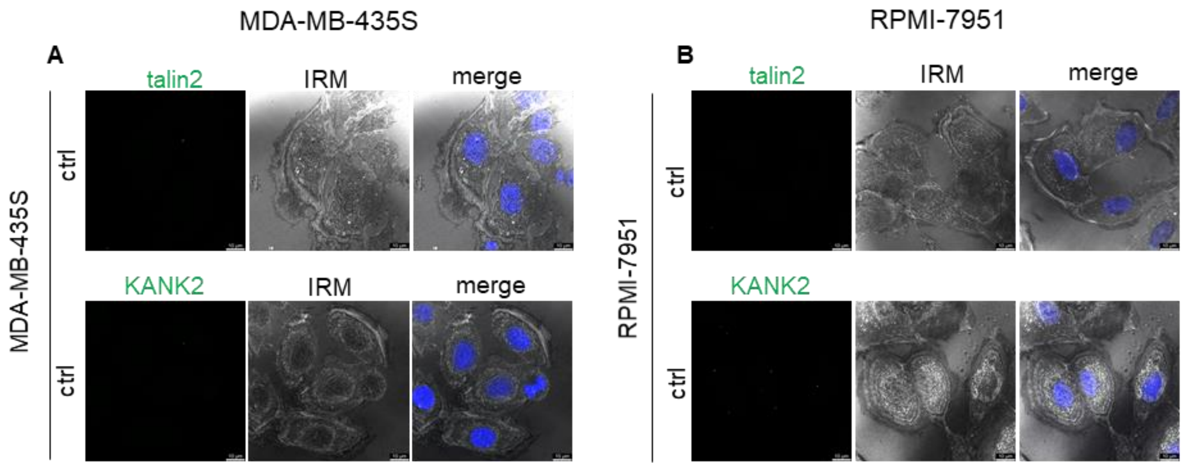

Supplementary Fig. S3 Control of antibody specificity for PLA assay. Forty-eight hours after seeding, cells were methanol fixed, and a PLA assay with anti-talin2 or anti-KANK2 primary antibody was performed. Presence of unspecific fluorescent signals was checked and IRM images were taken. Analysis was performed using TCS SP8 Leica. Scale bar = 10 µm.

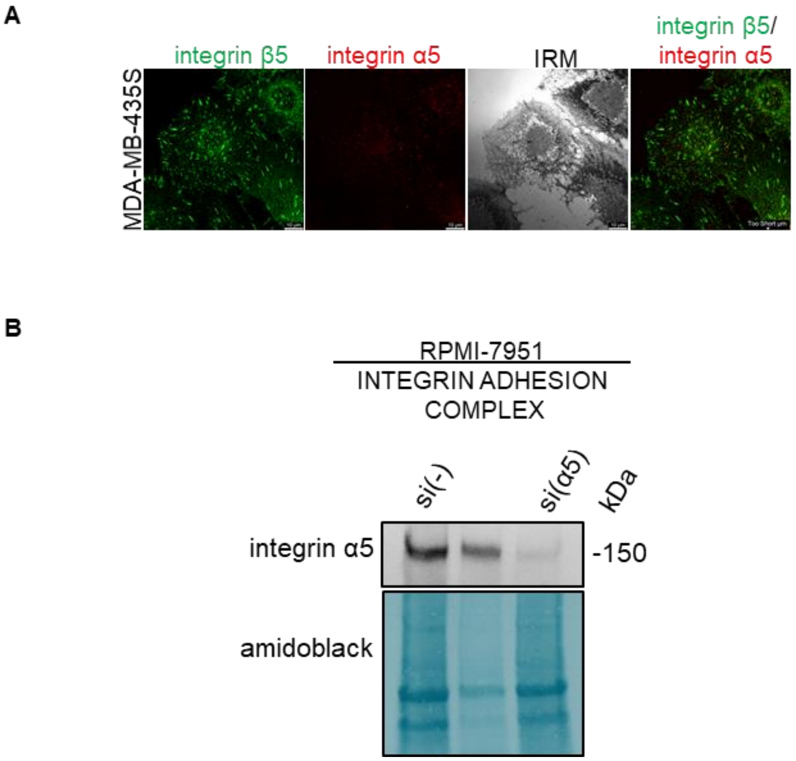

Supplementary Fig. S4 (A) MDA-MB-435S cells contain very little to none α5-positive adhesions. Forty-eight hours upon seeding on coverslips, cells were fixed with PFA, permeabilised and stained with anti-integrin β5 and anti-integrin α5 followed by Alexa-Fluor 488-conjugated antibody (green) or Alexa-Fluor 546-conjugated antibody (red), respectively. IRM images were taken. Analysis was performed using TCS SP8 Leica. Scale bar = 10 μm. (B) Validation of integrin α5 knockdown by specific siRNA. WB analysis of integrin α5 in RPMI-7951 cells transiently transfected with control siRNA (si(-)) versus integrin α5 -specific siRNA (si(α5)). IACs were isolated and WB analysis was performed. Amidoblack staining of the membrane was used as a loading control. The results presented are representative of at least three independent experiments yielding similar results.

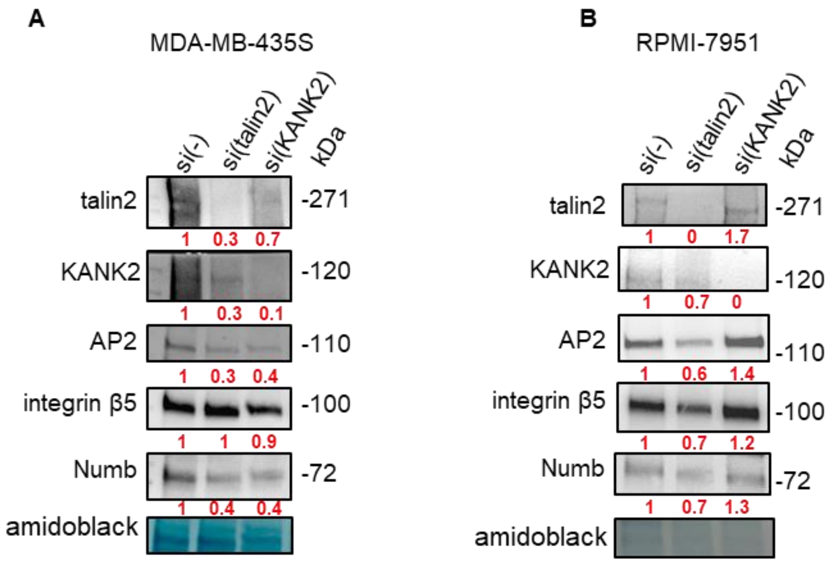

Supplementary Fig. S5 Validation of MS data upon knockdown of talin2 or KANK2 (A, B) WB analysis of RA proteins in MDA-MB-435S (A) and RPMI-7951 cells (B) transiently transfected with control siRNA (si(-)) versus talin2-specific siRNA (si(talin2)) or KANK2-specific siRNA (si(KANK2)). Two hours upon CytoD treatment, IACs were isolated and WB analysis was performed. Amidoblack staining of the membrane was used as a loading control. The results presented are representative of at least two independent experiments yielding similar results.
