# Supplementary material for: Regulation of reticular adhesions by KANK2 and talin2 in two melanoma cell lines
Source: Cell Commun Signal. 2026 Apr 24;24:338. doi: 10.1186/s12964-026-02904-1 (PMC13244953; doi:10.1186/s12964-026-02904-1)
Supplement: Supplementary file 3 — Supplementary Material 3. [file 12964_2026_2904_MOESM3_ESM.pdf]

Supplementary Material 2

Regulation of reticular adhesions by KANK2 and talin2 in two melanoma cell lines

Anja Rac[1,*] ORCID:0000-0001-8821-3059, Marija Lončarić[1,*] ORCID:0000-0002-5343-0368, Nikolina Stojanović[1,*,#] ORCID:0000-0002-7763-4154, Mahak Fatima[2] ORCID: 0000-0003-2780-0844, Mirna Rešetar[1], Dalibor Hršak[3] ORCID: 0000-0002-1462-7424, Jonathan D. Humphries[4] ORCID:0000-0002-8953-7079, Martin J. Humphries[2] ORCID:0000-0002-4331-6967, Andreja Ambriović-Ristov[1,#] ORCID:0000-0001-7784-2466

[1]Laboratory for Cell Biology and Signalling, Division of Molecular Biology, Ruđer Bošković Institute, Zagreb, Croatia; [2]Manchester Cell-Matrix Centre, Faculty of Biology, Medicine & Health, University of Manchester, Manchester, United Kingdom; [3]Laboratory for Computational Biology and Translational Medicine, Division of Electronics, Ruđer Bošković Institute, Zagreb, Croatia; [4]Department of Life Science, Manchester Metropolitan University, Manchester, United Kingdom

*equal contribution

[#]corresponding authors: Nikolina.Stojanovic@irb.hr, Andreja.Ambriovic.Ristov@irb.hr

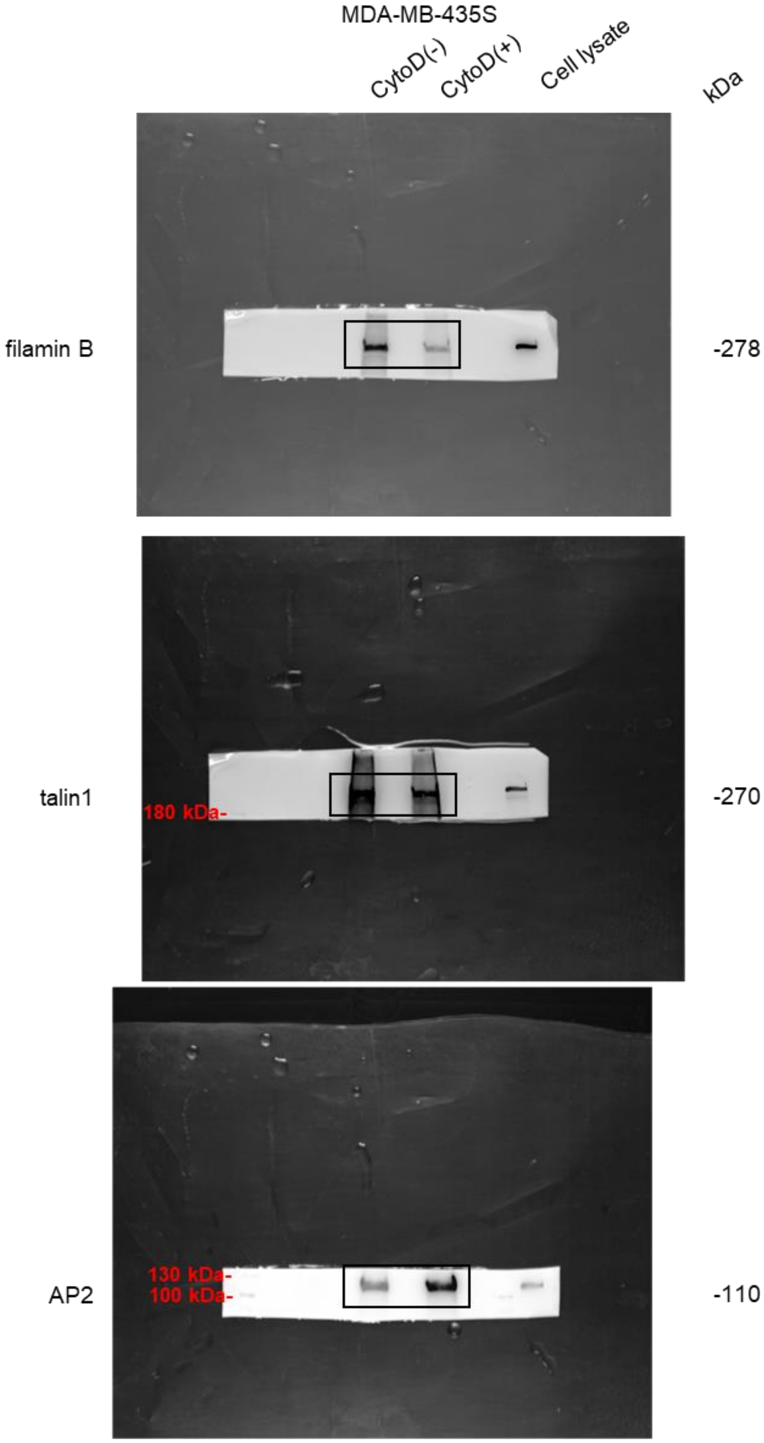
MDA-MB-435S
CytoD(-)
CytoD(+)
Cell lysate
kDa
filamin B
-278
talin1
180 kDa-
-270
AP2
130 kDa-
100 kDa-
-110

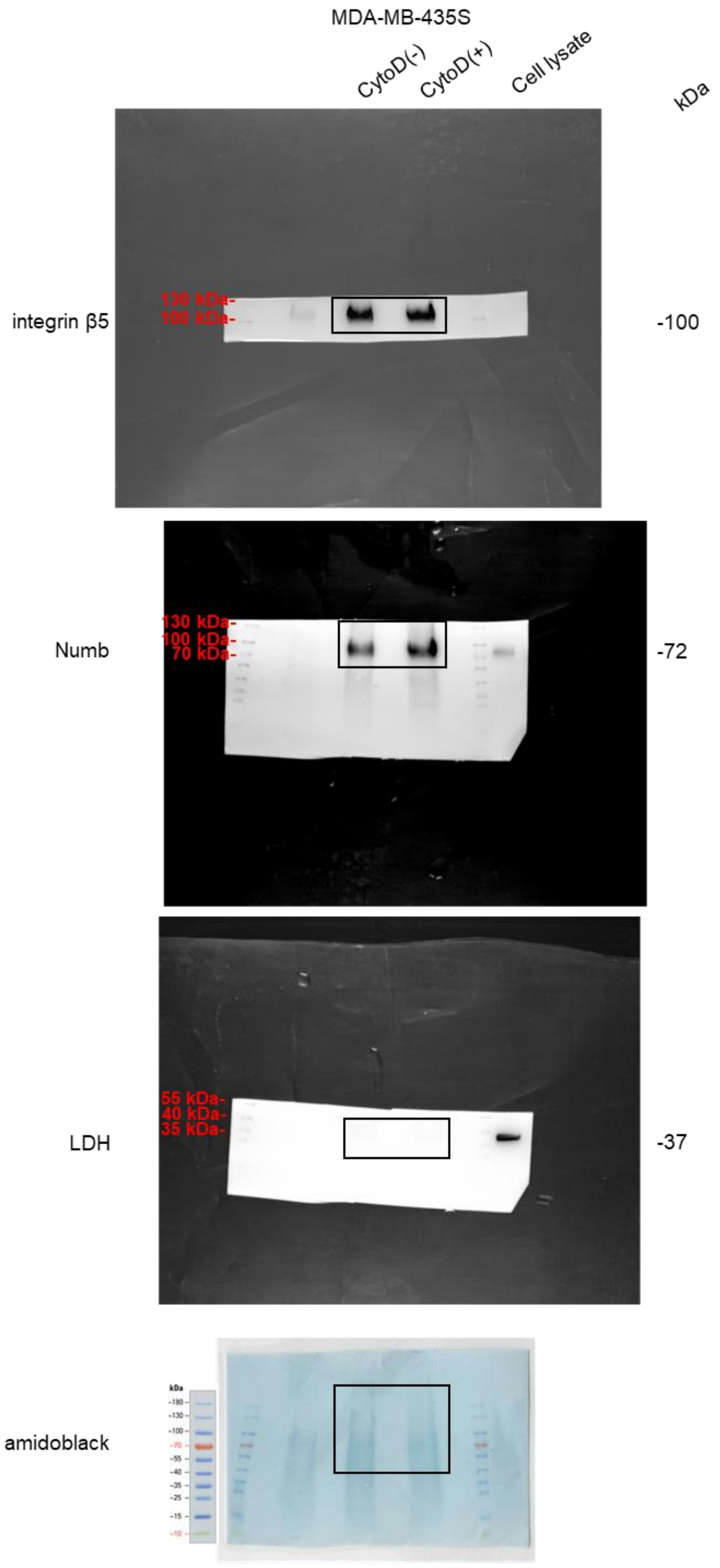

Supplementary Fig. S6 Full images of the blots in Fig. S2A. Images were obtained using Uvitec Alliance Q9 mini, which directly scanned membranes developed with ECL reagents.

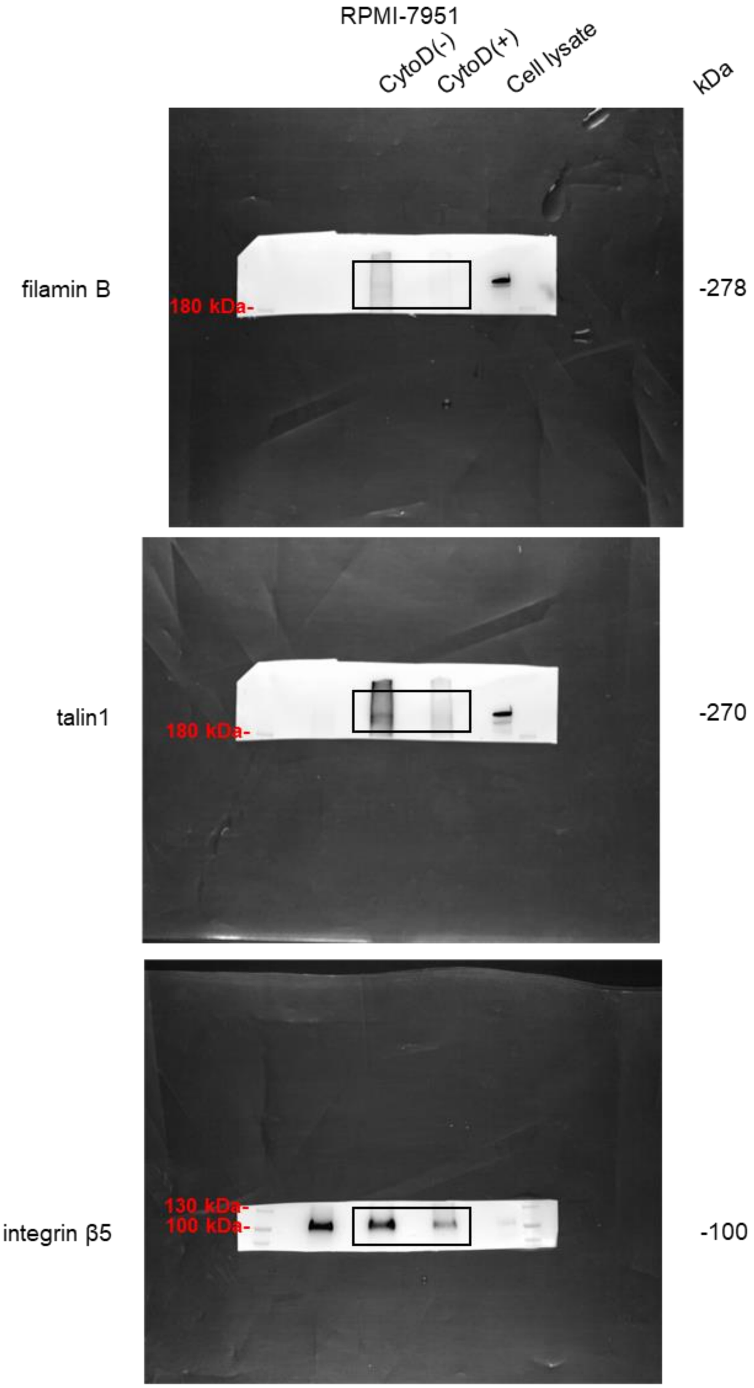
RPMI-7951
CytoD(-)
CytoD(+)
Cell lysate
kDa
filamin B
180 kDa-
-278
talin1
180 kDa-
-270
integrin β5
130 kDa-
100 kDa-
-100

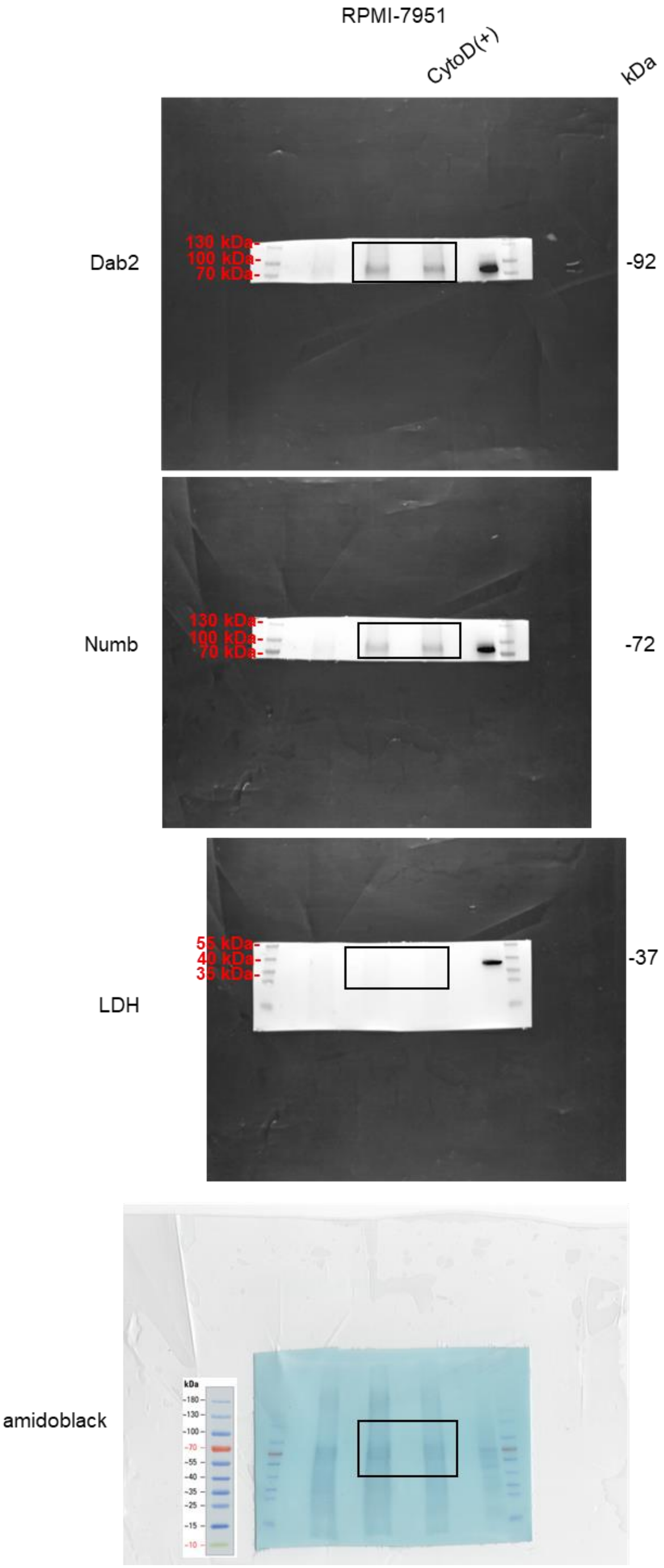

Supplementary Fig. S7 Full images of the blots in Fig. S2B. Images were obtained using Uvitec Alliance Q9 mini, which directly scanned membranes developed with ECL reagents.

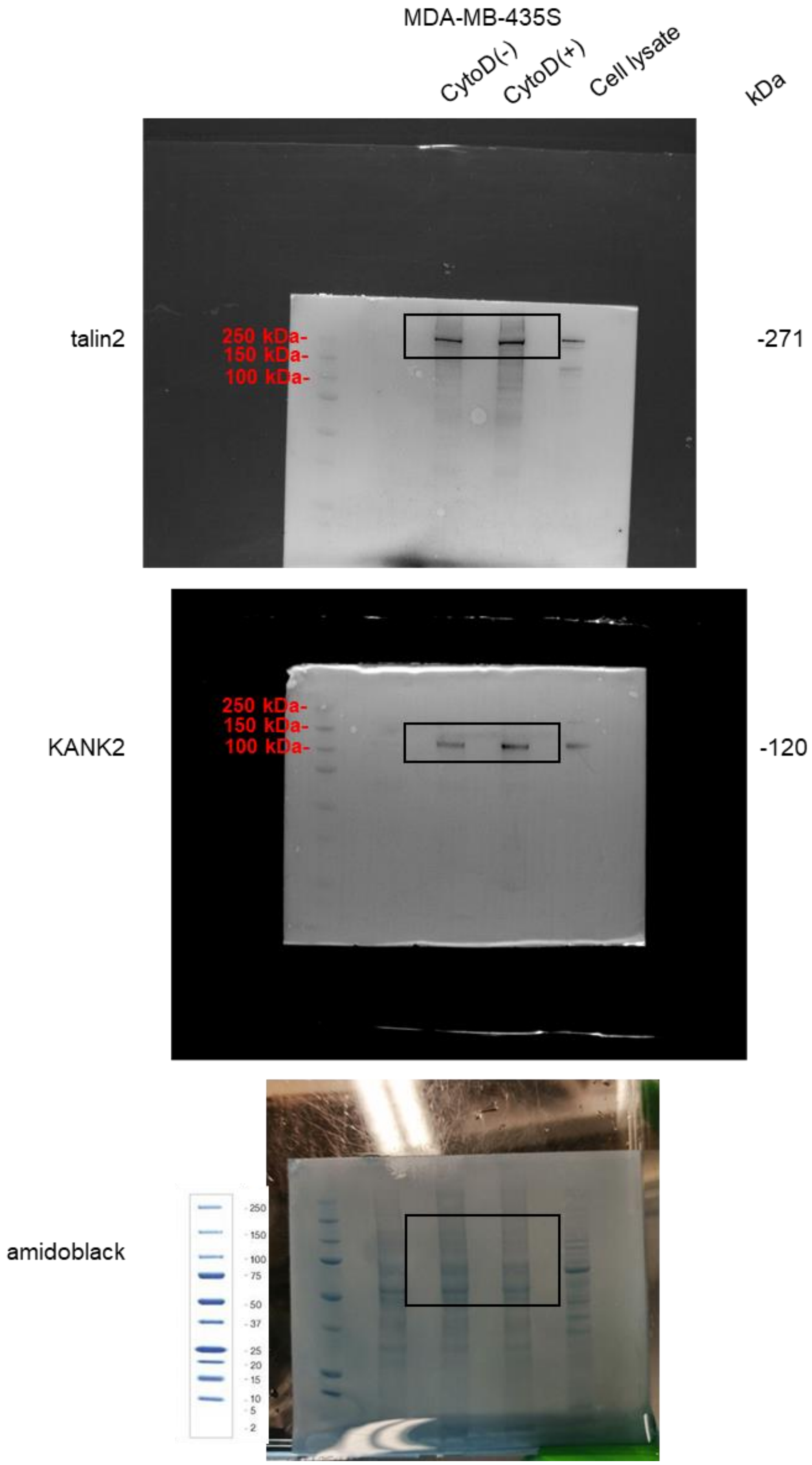

Supplementary Fig. S8 Full images of the blots in Fig. S2C. Images were obtained using Uvitec Alliance Q9 mini, which directly scanned membranes developed with ECL reagents.

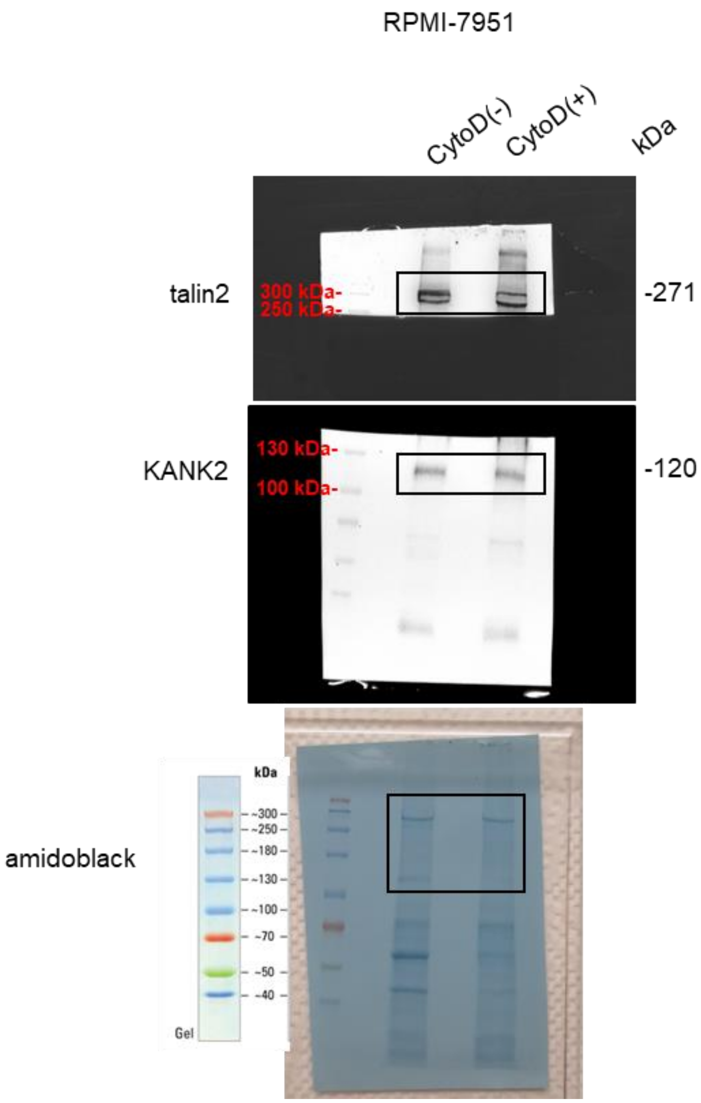

Supplementary Fig. S9 Full images of the blots in Fig. S2D. Images were obtained using Uvitec Alliance Q9 mini, which directly scanned membranes developed with ECL reagents.

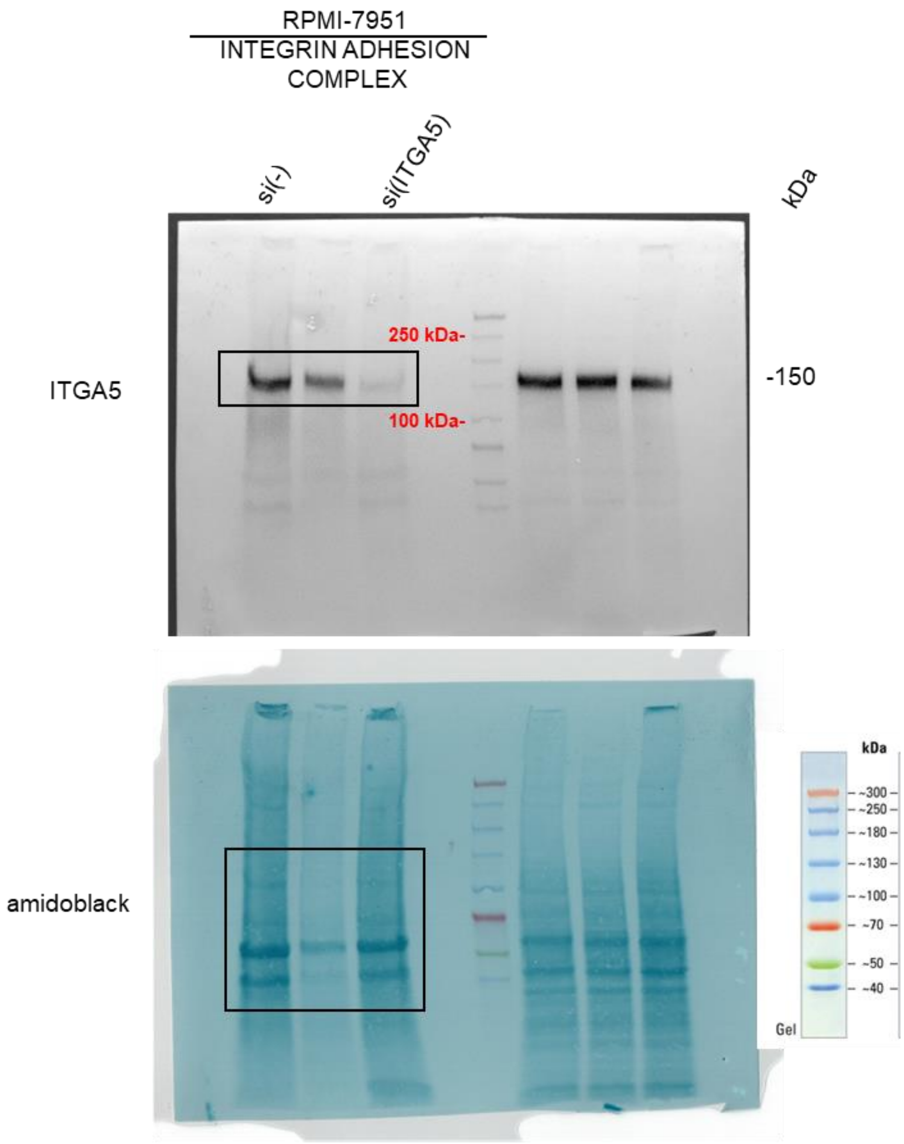

Supplementary Fig. S10 Full images of the blots in Fig. S4B. Images were obtained using Uvitec Alliance Q9 mini, which directly scanned membranes developed with ECL reagents.

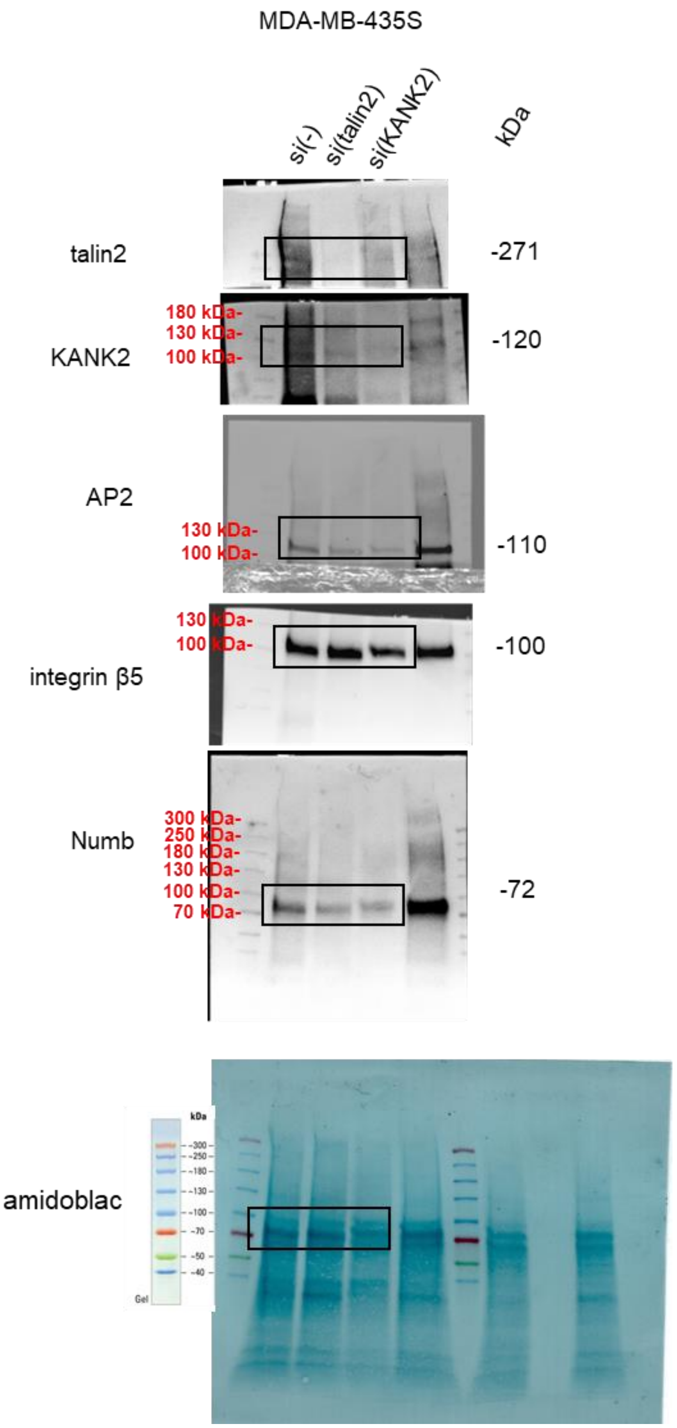

Supplementary Fig. S11 Full images of the blots in Fig. S5A. Images were obtained using Uvitec Alliance Q9 mini, which directly scanned membranes developed with ECL reagents.

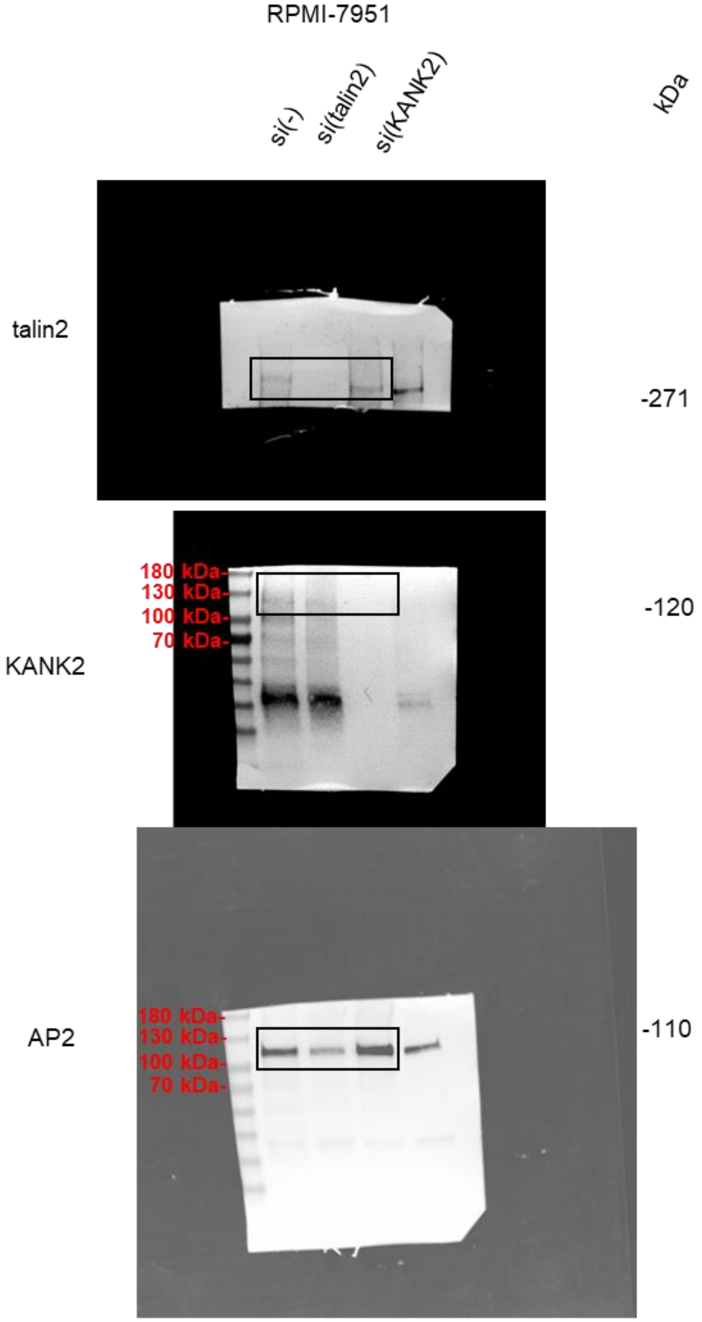
RPMI-7951
si(-)
si(talin2)
si(KANK2)
kDa
talin2
-271
180 kDa-
130 kDa-
100 kDa-
70 kDa-
KANK2
-120
180 kDa-
130 kDa-
100 kDa-
70 kDa-
AP2
-110

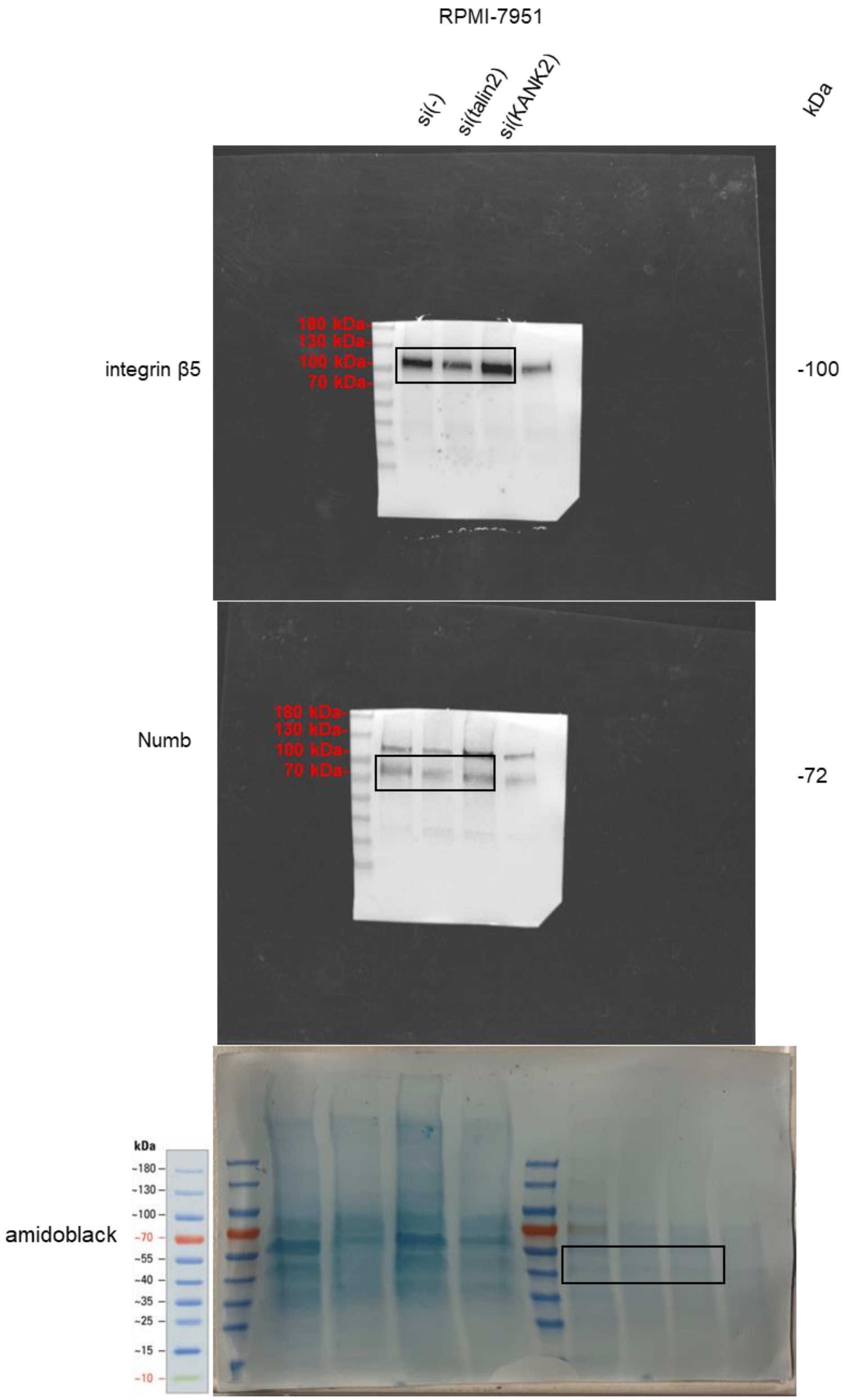

Supplementary Fig. S12 Full images of the blots in Fig. S5B. Images were obtained using Uvitec Alliance Q9 mini, which directly scanned membranes developed with ECL reagents.
